# A Comprehensive Analysis of *CSN1S2* I and II Transcripts Reveals Significant Genetic Diversity and Allele-Specific Exon Skipping in Ragusana and Amiatina Donkeys

**DOI:** 10.3390/ani14202918

**Published:** 2024-10-10

**Authors:** Gianfranco Cosenza, Alfredo Pauciullo

**Affiliations:** 1 Department of Agriculture, University of Naples Federico II, 80055 Portici, NA, Italy; 2 Department of Agricultural, Forest and Food Sciences, University of Turin, 10095 Grugliasco, TO, Italy; 3 National Research Council of Italy, Institute of Animal Production in the Mediterranean Environment, Piazzale Enrico Fermi 1, 80055 Portici, NA, Italy

**Keywords:** donkeys, Ragusana and Amiatina breeds, αs2-casein, *CSN1S2*, transcript analysis

## Abstract

**Simple Summary:**

In recent decades, interest in the use of donkey milk for human nutrition has increased, since it may represent a natural substitute for cow’s milk for children affected by milk protein allergies. The functional peculiarities of donkey milk are mainly linked to its casein content comparable to that of human milk. This study provides a thorough analysis of transcript isoforms generated by two αs2-casein-encoding genes (*CSN1S2* I and *CSN1S2* II) in donkeys and a detection of significant genetic diversity at both loci along with sequence comparisons across species. In particular, a key mutation affecting exon 17 splicing in *CSN1S2* I was identified, and a genotyping method was developed. These data represent an important step in the understanding of the expression regulation of these genes in donkeys and a useful tool for the genetic improvement of donkey milk production that fulfils special consumer requirements.

**Abstract:**

The αs2-casein is a phosphoprotein secreted in the milk of most mammals, and it is the most hydrophilic of all caseins. Contrary to genes found in ruminants, in donkeys two different encoding genes for donkey αs2-casein (*CSN1S2* I and *CSN1S2* II) have been identified. However, unlike in ruminants, the variability at these loci has not been characterized in detail in donkeys until now. In this study, we analyze the transcript profile of the donkey *CSN1S2* I and *CSN1S2* II genes, and we identify and describe the variability of these loci in the Ragusana and Amiatina breeds reared in Italy. The analysis of the *CSN1S2* I Reverse Transcriptase-Polymerase Chain Reaction (RT-PCR) products and subsequent sequencing showed, in addition to correctly spliced mRNA, seven other minor mRNAs resulting from differential splicing events involving, in various combinations, entire exons (4, 5, 6, and 11), parts of exons (5′ or 3′ end of exon 17), or the recognition of intronic sequences as an exon (exon 12′). Similarly, the transcription analysis of the *CSN1S2* II gene revealed a remarkable variability in splicing events, mainly concerning the alternative insertion of an extra exon 7 (named 7′); the first 33 bp of exon 13; or the alternative skipping of exons 9, 10, 11, 12, and 15, and their combinations. At the mRNA level for *CSN1S2* I, seven SNPs were observed, five of which led to amino acid changes: p.T73>A, p.I109>V, p.I130>V, p.I146>T, and p.D217>Y. Similarly, nine SNPs were observed at the *CSN1S2* II locus, seven of which are non-synonymous: p.L63>F, p.H70>Q, p.D90>N, p.129A>T, p.H131>Y, p.E144>G, and p.F157>S. In addition, the DNA sequencing of exon 17 and flanking introns of the *CSN1S2* I gene revealed a G>A transition at the splice acceptor site of *CSN1S2* I exon 17 (FM946022.1:c.375-1G>A), resulting in an allele-specific skipping of the first 15 nucleotides of this exon, which encode the peptide 176NKINQ180, and the recognition of an in-frame cryptic splicing acceptor site: arAACAAAATCAACCAG. A genotyping method based on restriction fragment length polymorphism (*XbaI* PCR-RFLP) was set up for this SNP. In the total population studied (105 Ragusana and 14 Amiatina donkeys), the A allele had a frequency of 0.2437 with no evidence of deviation from the Hardy–Weinberg equilibrium. This study adds new knowledge regarding the genetic variability of αs2-caseins in donkeys and may contribute significantly to the genetic improvement of milk production for this species.

## 1. Introduction

The domestic donkey (*Equus asinus*) is a quadrupedal mammal belonging to the order of odd-toed ungulates (*Perissodactyla*) and sub-order of horse-like (*Hippomorpha*). It is part of the horse family (*Equidae*), which also includes the horse (*Equus caballus*) and the zebra (*Equus zebra*). This family includes the genus horse (*Equus*).

Although the donkey species continues to maintain great importance in many developing countries, technological evolution, the economic development of many rural areas, and the spread of agricultural machinery are making this animal increasingly less useful to its traditional “employer”. Consequently, especially in industrialized countries like Italy, it increasingly risks the fate of its wild ancestors. In the 1950s, there were almost a million donkeys in Italy, but their population has drastically decreased over time. It was only in 2000 that a rapid recovery began, saving many autochthonous breeds such as the Asinara, Pantesco, Grigio Siciliano, Romagnolo, Amiatina, Sardo Grigio, Martina Franca, and Ragusana [1,2] from extinction. One reason for this is the growing interest in the use of donkey milk for human nutrition, leading to this species now being considered a minor dairy species. Different studies have been performed to evaluate the safety of donkey milk in patients allergic to cow’s milk. The results of these studies were consistent, showing that more than 80% of the examined cow’s milk allergic patients tolerated donkey milk after an oral food challenge [3]. In addition, donkey milk is efficient in releasing anti-inflammatory interleukins, upregulating the immune response in aged hosts, preventing atherosclerosis, and acting as an anti-proliferative and anti-tumor agent [4]. Furthermore, the parapharmaceutical and cosmetic industries are particularly interested in formulating products based on donkey milk [5], whose therapeutic and aesthetic properties have been known since ancient times. 

The functional peculiarities of donkey milk are mainly linked to its protein content. In fact, donkey milk is characterized by a lower content of caseins and whey proteins compared to ruminants’ milk, and its quantity of casein is comparable to that of human milk. In particular, the average content of caseins (34.61%) and whey proteins (49.80%), with a casein to whey protein ratio of 0.69, shows remarkable differences in comparison with bovine and other ruminant milks. The casein fraction in donkey milk (5.12 mg/mL) is mainly represented by αs1-CN (1.82 mg/mL) and β-CN (2.77 mg/mL), as well as smaller amounts of αs2-CN (3.68 × 10^−1^) and κ-CN (1.42 × 10^−1^) [6].

However, despite the growing interest in this species and its production, studies on the quantitative and qualitative aspects of donkey milk production are still limited compared to those conducted on the milk of other species, such as ruminants of zootechnical interest. Only recently have several studies been carried out to characterize its protein components and the genetic variability of the main genes coding for the different casein and whey protein fractions [6,7,8,9,10,11,12,13,14,15].

The proteomic approach has allowed characterization of the casein compositional heterogeneity due to post-translational modifications, like phosphorylation (αs1, αs2, and β-CN) and glycosylation (κ-CN) [7,8].

In particular, αs2-CN shows great heterogeneity due to variable degrees of phosphorylation [7,8,16,17]. The complex polymorphism of donkey αs2-CN is also characterized by the identification of two different *CSN1S2* genes, called *CSN1S2* I and *CSN1S2* II, so far characterized only at the cDNA level. The first, spanning over a fragment of 1016 bp, is constituted by 19 exons, and it encodes for a protein of 221 amino acids (called αs2-CN-I); on the other hand, the second is constituted by 16 exons and probably originated by gene duplication [12]. Both genes, similarly to the equine species, are closely associated and map on chromosome 3 (gene IDs 106835119 and 106828076, respectively, for *CSN1S2* I and *CSN1S2* II, https://www.ncbi.nlm.nih.gov/gene/ accessed on 9 October 2024). Contrary to what happens in humans, but similar to rabbit, the *CSN1S2* II gene seems to be functional in donkeys, leading to the effective translation of mRNA into the αs2-CN-II protein [7].

Of the two genes, the one that has been relatively more investigated is *CSN1S2* I, characterized at the transcript and/or proteomic level in Ragusana breed donkeys [6,12,16,17]. More recently, both genes have been characterized using a combination of molecular biology (cDNA sequencing) and proteomic tools in the Amiatina breed [7].

The research conducted by these authors highlights significant genetic variability and the existence of different αs2-CN splicing isoforms, although the results are not always directly comparable, likely due to the two different breeds investigated.

Therefore, the primary aim of the present study was to comprehensively characterize the transcripts of the *CSN1S2* I and *CSN1S2* II genes in the Ragusana breed, analyze splicing and skipping events, investigate the genetic diversity of these two genes, and propose an evolutionary phylogenetic analysis to understand *CSN1S2* gene differentiation among representative species of the orders *Perissodactyla*, *Cetartiodactyla*, and *Carnivora.* The Amiatina donkey has been used as a comparison breed.

## 2. Materials and Methods

### 2.1. Donkey Breeds 

The Ragusana donkey, a native Sicilian breed from southern Italy, is one of the most productive breeds reared in Italy. It is used for recreational activities, trekking, and onotherapy, as well as for its notable milk production. In fact, with an average daily milk yield of 1.64 kg, it is more productive than other Italian breeds such as Amiatina and Martina Franca [18].

The Amiatina donkey is an endangered breed from Tuscany (central Italy), with additional animal units in Liguria and in southern Italy (Campania region). Historically, this breed was used as a pack animal on farms and in mines. Today, it is used for trekking, onotherapy, and milk production with an average daily milk yield of 0.7 kg [18,19].

### 2.2. mRNA Samples

For this study, 8 unrelated female donkeys of the Ragusana breed were used. They were of comparable age (approximately 6 years old); fed the same type of feed (hay and concentrate); and were at similar diet, feeding level, and lactation stage (about 60 days). The donkeys were kept in individual indoor stalls and reared on the same breeding farm located in southern Italy. Total RNA was extracted from somatic cells present in the milk using Nucleospin^®^ RNA Plus XS (Macherey-Nagel, Düren, Germany). All milk samples used for the analyses were collected by authorized personnel during the periodic veterinary control.

### 2.3. DNA Samples 

All the samples used in this study were obtained from previous research and are part of collections of the University of Torino (northern Italy) and University of Naples (southern Italy). Specifically, 105 DNA samples were originally isolated from individual blood samples of Ragusana donkeys, and 14 samples were from Amiatina donkeys, reared in different Italian regions. These individual blood samples were collected during routine prophylaxis by official veterinarians from the Local Sanitary Units (ASLs) of the Ministry of Health. Therefore, approval from the Animal Care and Use Committee was not necessary. 

### 2.4. Primer Design, RT-PCR Conditions for Amplification, and Cloning of the Donkey CSN1S2 I and CSN1S2 II cDNAs

Primer pairs, purchased from Eurofins (Eurofins Genomics, Ebersberg, Germany), were designed using published *Equus asinus* nucleotide (cDNA) sequences (Supplementary Material, Table S1).

Reverse transcriptions (RTs) for the amplification of the cDNA of the donkey *CSN1S2* I and *CSN1S2* II genes were performed by using the ImProm-II™ Reverse Transcriptase and oligo dT for priming, following the manufacturer’s instruction protocol (Promega, Madison, WI, USA). 

The 100 μL PCR reaction mix for the amplification of both cDNAs comprised 20 μL of RT reaction product; 50 mM KCl; 10 mM Tris–HCl (pH 9.0); 0.1% Triton X-100; 2 mM MgCl_2_; 10 pmoL of each primer, dNTPs each at 0.2 mM; and 5 U of Taq DNA Polymerase (Promega, Madison, WI, USA). The thermal profile of the PCR amplification consisted of 39 cycles: the first cycle involved a denaturation step at 97 °C for 2 min, an annealing step at 57 °C (for *CSN1S2* I) or 50 °C (for *CSN1S2* II) for 30 s, and an extension step at 72 °C for 1 min and 30 s. The next 37 cycles were performed under the following conditions: 94 °C for 30 s, 57 or 50 °C for 30 s, and 72 °C for 1 min and 30 s. In the 39th cycle, the extension step was carried out at 72 °C for 10 min.

The amplified products were first analysed by electrophoresis on a 2% agarose gel in TBE 0.5X buffer, stained with SYBR green nucleic acid stain (Lonza Rockland Inc., Rockland, ME, USA), and then cloned using the TOPO Cloning Reaction Kit (Invitrogen, Life Technologies Inc., Carlsbad, CA, USA), following the manufacturer’s instructions.

### 2.5. Screening of Clones by PCR and Sequencing

The screening of the clones was accomplished by PCR using the same forward and reverse primers used for the initial cDNA amplification. The reaction took place in 25 μL of mix, which included 0.5 μL of overnight fluid cultures of transformed cells, 50 mM KCl; 10 mM Tris–HCl (pH 9.0); 0.1% Triton X-100; 3 mM MgCl_2_; 5 pmol of each primer, dNTP 200 μM each; 2.5 U of Taq DNA Polymerase; and 0.04% BSA. The amplification program began with a cell lysis and nuclease inactivation step at 94 °C for 10 min. Subsequently, 30 reaction cycles were performed as indicated in the previous paragraph. Plasmids from positive colonies were sequenced on both strands by Eurofins Genomics (Ebersberg, Germany). 

### 2.6. Genomic DNA Sequencing

The donkey genome sequence (GenBank acc. no. JADWZW020000003.1, region: 152933458 to 152933994, chromosome 3) was used to design primers (Table S1) for PCR amplification and sequencing of the DNA region spanning exon 17 and the 3′-flanking regions of the *CSN1S2* I gene.

The reaction mix volume was 25 μL and was composed of 100 ng of genomic DNA; a 1X Green GoTag Flexi Buffer; 1.5 mM of MgCl_2_; 200 μM of each dNTP, 10 pmol of each primer; and 1 U of GoTaq^®^ G2 Flexi DNA Polymerase (Promega, Madison, WI, USA).

The amplification programs consisted of 31 cycles. The first cycle was characterized by denaturation at 97 °C for 2 min, annealing at 54.5 °C for 45 s, and an extension step at 72 °C for 1 min. The next 30 cycles involved denaturation at 94 °C for 45 s, annealing at 54.5 °C for 45 s, and extension at 72 °C for 1 min, with the exception that in the last cycle the extension time was prolonged to 10 min. All amplicons were sequenced on both strands by Eurofins Genomics (Ebersberg, Germany).

For the genomic sequencing of the donkey *CSN1S2* I gene, we selected 16 test samples: the same 8 Ragusana subjects used for transcript analysis plus 8 randomly chosen Amiatina donkeys.

### 2.7. Genotyping of Donkey at CSN1S2 I Locus by XbaI PCR-RFLP

A genotyping method based on PCR-RFLP (Restriction Fragment Length Polymorphism) was developed to screen the FM946022.1: c.375-1G>A transition at the splice acceptor site of the donkey *CSN1S2* I exon 17 in the population.

The DNA fragment of 558 bp was amplified using the primers, reaction mix, and thermal conditions described above for genomic DNA sequencing.

Digestion of 6 μL of each PCR amplification was performed with 10 U of *Xba*I endonuclease (Promega, Madison, WI, USA) for 5 h at 37 °C, following the supplier’s directions for buffer conditions. PCR and digestion products were analysed directly on 3% agarose gels in TBE buffer 0.5X and stained with SYBR green nucleic acid stain (Lonza Rockland Inc., Rockland, ME, USA).

The entire panel of 119 donkey DNA samples was genotyped.

### 2.8. Bioinformatics and Statistical Analysis

Allele frequencies were calculated by simple allele counting. Possible deviations of genotypic frequencies from expectations were tested by a chi-square test to verify if the population was in the Hardy–Weinberg equilibrium. Homology searches, comparisons among nucleotide and amino acid sequences, and multiple alignments were accomplished using Dnasis Pro 3.0 (Hitachi Software Engineering Co., Tokyo, Japan).

## 3. Results and Discussion

### 3.1. Analysis of the Transcripts

#### 3.1.1. *CSN1S2* I

Amplicons obtained by the retro-transcription of eight donkey mRNA samples were cloned, screened, and sequenced.

**Figure 1:**
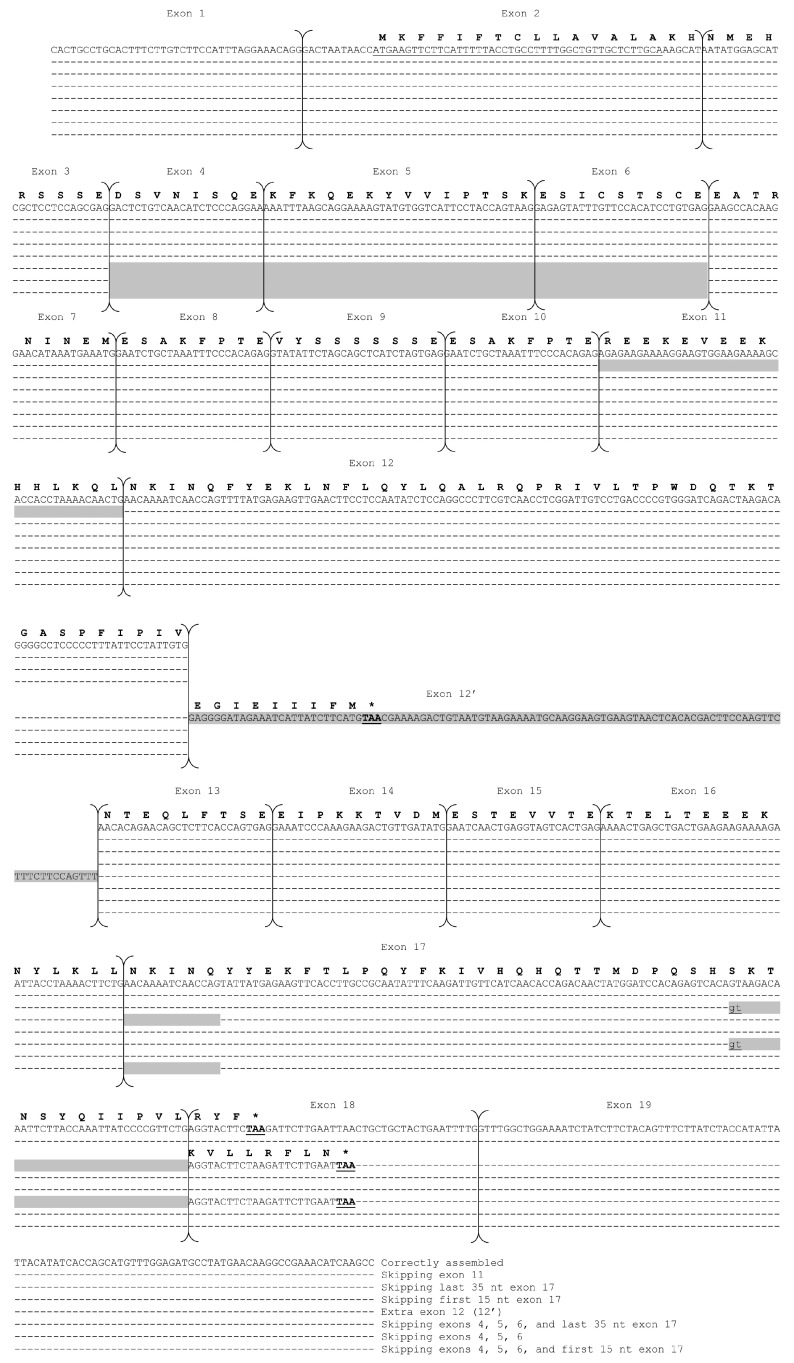
Nucleotide sequences of cDNA clones coding for αs2-CN-I. Dashes represent identical nucleotides to those in the upper lines. The deduced protein sequence is shown above each corresponding nucleotide sequence. The large arrows indicate exons of the mRNA as deduced from known splice junctions of the donkey *CSN1S2* I gene [12]. Exon numbering (above the blocks) follows the method of Cosenza et al. [12]. Exon 12′ is an additional exon compared to the donkey *CSN1S2* I cDNA sequence (FM946022.1); therefore, it is numbered with a prime (′). Gray boxes depict skipped exons or sequences absent from the sequenced cDNA clones. The stop codon is indicated by an asterisk (*). The exon 17 gt cryptic splice site is in bold. Alignment was performed using DNAsis pro Software v2.0 (Hitachi).

The analysis of the cloned RT-PCR fragments of the *CSN1S2* I gene was performed on a total of 160 positive clones (about 20 per sample). Sequencing results of 80 randomly chosen clones (10 per sample) showed the presence of at least 8 cDNA populations (Table 1; Figure 1).

The most represented population was that correctly assembled encoding for a full-length donkey αs2-CN I of 221 amino acids. According to Cosenza et al. [12], this cDNA is composed of 19 exons, ranging in size from 24 bp (exons 4, 8, and 15) to over 100 bp (exon 19). The signal peptide (MKFFIFTCLLAVALA) is coded from the 12th to the 56th nucleotide of the exon 2, while the stop codon (TAA) is located at the 10th–12th nucleotide of the exon 18.

The remaining seven transcripts are the results of differential splicing events involving, in various combinations, entire exons (4, 5, 6, and 11) or parts of exons (5′ or 3′ end of the 17th exon), or the recognition of intronic sequences as an exon (exon 12′) (Table 1; Figure 1). 

In detail, the alternative skipping of the first 15 nucleotides of exon 17 (AACAAAATCAACCAG, Supplemental Figure S1) is responsible for the deletion of the pentapeptide ^176^NKINQ^180^, which appears to be constitutively spliced in the αs2-CN from mares [17].

This seems to be due to a G>A transition that alters the canonical splice acceptor site in equine species, leading to the recognition of a second in-, frame splice acceptor site (nucleotides 14–15) [7]. We observed the alternative skipping of the first 15 nucleotides of exon 17 either alone or in conjunction with the simultaneous skipping of exons 4, 5 and 6, similarly to what has been reported by Saletti et al. [17] and Cunsolo et al. [16] in the same breed. The first 15 bp of exon 17 are a perfect duplication of the first 15 nucleotides of exon 12 (coding for the pentapeptide ^92^NKINQ^96^). It is likely that exon 12 and exon 17, both 129 bp in length, are at least partially the result of an internal duplication, similar to that of exons 11 and 16 (Supplemental Figure S2).

Exon duplication is a common event in the evolution of the *CSN1S2* and *CSN1S1* genes. The presence of many small exons of similar size suggests that duplication events have contributed to the exon structure of these genes in mammals [20].

For example, in donkey *CSN1S2* I, exons 8 and 10, along with the flanking regions, are perfectly duplicated (Supplemental Figure S2). A similar duplication event was observed also in Old-World Camels’ *CSN1S2* cDNA where exons 8 and 11 shared 22 and 17 out of 24 bp (ESAEVTPE and ESTEVTPE). These two exons in camels corresponded to exon 9 in the cattle *CSN1S2* sequence, where no such duplication occurred [21].

Similarly, duplications of exons or the recognition of introns as coding sequences also characterize the *CSN1S2* gene in bovids [20,22]. A second event involves exon 17 and the alternative skipping of its 3′ extremity (35 nucleotides) (Supplemental Figure S3) either together with or independent of the concurrent absence of exons 4, 5, and 6 (Figure 1; Table 1). 

The deletion observed at the 3′ end of exon 17 reveals a cryptic splice site (casual usage of cryptic splice sites), disrupting the coding triplet of the αs2-casein reading frame and causing a frameshift of the termination codon (TAA), which is normally established with the third codon of exon 18, now changed to a TAA stop codon 26 nucleotide downstream in exon 18. Such an event would lead to the translation of a sequence of the exon 18, which is normally only transcribed. As a consequence, this mRNA retains an open reading frame despite the frameshift and would translate an αs2 lacking the peptide ^207^SKTNSYQIIPVLRYF^221^ but with a new C-terminal sequence: ^207^KVLLRFLN^214^ (Figure 1).

Transcripts characterized by this event in the donkey species were also observed by Saletti et al. [17], Cunsolo et al. [16], and Auzino et al. [7]. However, Saletti et al. [17] and Cunsolo et al. [16] reported only the deletion of the peptide YQIIPVL. 

A similar event was also observed at the 3′ end of the exon 15 of the *CSN1S2* gene in camelids (corresponding to exon 17 in donkeys), where an isoform results from an alternative splicing event of the decapeptide VKAYQIIPNL due to the identification of a cryptic splice site, although the reading frame is not altered (Supplemental Figure S3) [21,23]. 

Finally, transcripts have been observed that are characterized by the presence of an extra exon of 105 nucleotides, which, being skipped between exons 12 and 13, is numbered as 12′ (Figure 1). This is likely an intronic sequence that is occasionally recognized as an exon.

The sequence of this extra exon, 12′, and its flanking regions, i.e., the splicing sites, is perfectly conserved also in the horse and zebra *CSN1S2* I gene (Supplemental Figure S4). Thus, it is very likely that a small proportion of the mRNAs in these species also contain this exon.

Due to the presence of a TAA codon between nucleotides 28 and 30 of the extra exon, this transcript is expected to translate into a 143 amino acid protein ending with the nonapeptide EGIEIIIFM. However, since this is a premature translation termination, it may lead to rapid mRNA degradation by nucleases, resulting in a low level of such messengers in the cytoplasm. This process, known as non-sense-mediated RNA decay (NMRD), has already been described in goats for other casein-encoding genes, such as *CSN2*, *CSN1S1,* and *CSN1S2* [24,25,26].

Finally, the alternative skipping of exons 4, 5, and 6 observed in the present study was also reported by Cunsolo et al. [16] and Auzino et al. [7] (Table 1) and should therefore be considered constitutive, allele-independent events that occur during the maturation of the pre-mRNA of the donkey *CSN1S2* I gene.

#### 3.1.2. *CSN1S2* II

**Figure 2:**
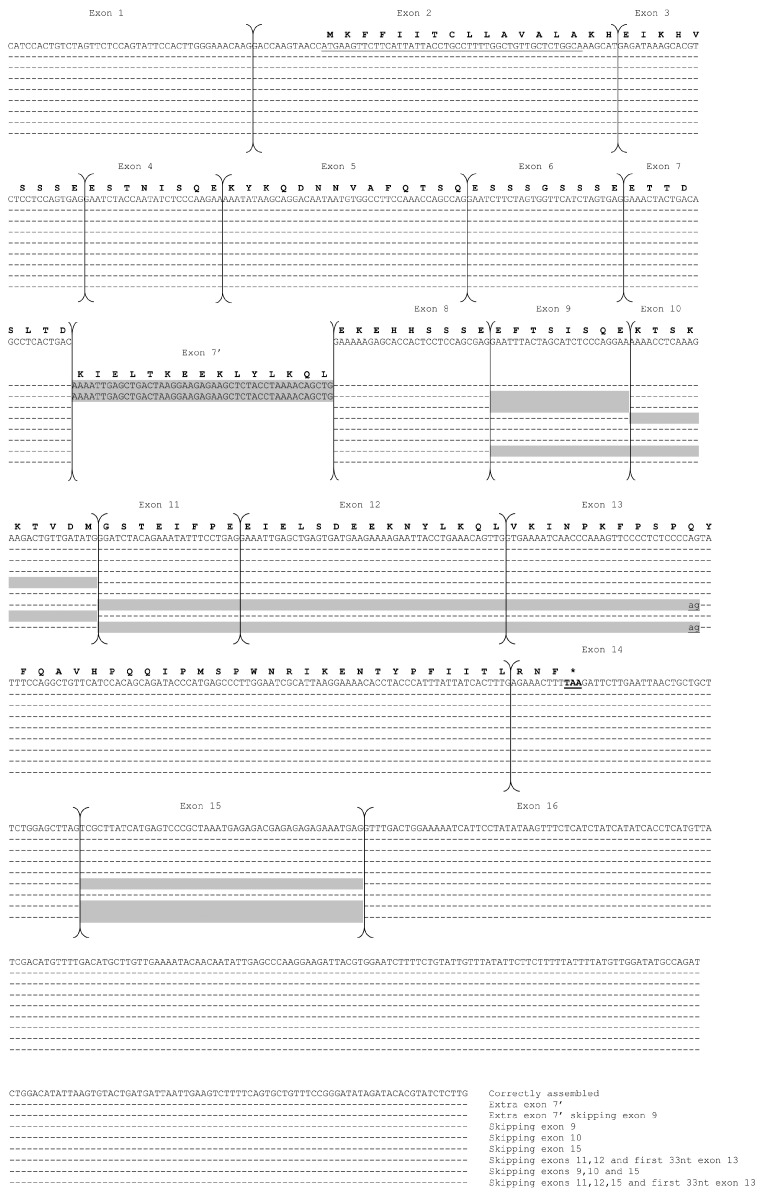
Nucleotide sequences of cDNA clones coding for αs2-CN-II. Dashes represent identical nucleotides to those in the upper lines. The deduced protein sequence is shown above each corresponding nucleotide sequence. The large arrows indicate exons of the mRNA as deduced from known splice junctions of the donkey *CSN1S2* II gene [12]. Exon numbering (above the blocks) follows the method of Cosenza et al. [12]. Ex 7′ is an additional exon compared to the donkey *CSN1S2* II cDNA sequence (FN298386.2); therefore, it is numbered with a prime (′). Gray boxes depict skipped exons or sequences absent from the sequenced cDNA clones. The stop codon is indicated by an asterisk (*). The exon 13 gt cryptic splice site is in bold. Alignment was performed using DNAsis pro Software v2.0 (Hitachi).

The screening of 160 positive clones (20 from each sample) of the *CSN1S2* II gene and the subsequent sequencing of about 60 randomly chosen clones revealed at least nine different populations of mRNA. The most represented population corresponds to a correctly assembled transcript encoding a mature donkey αs2-CN II of 142 amino acids. The remaining transcripts are characterized by the alternative skipping of individual exons (7′, 9, 10, or 15) or the simultaneous skipping of exons 9, 10, and 15; exons 11, 12, and the first 33 bp of exon 13; exons 11, 12, the first 33 bp of exon 13, and exon 15; or exon 9 with the presence of exon 7′ (Table 2, Figure 2).

According to Cosenza et al. [12], the correctly assembled mRNA consists of 16 exons, with sizes ranging from 24 bp (exons 7, 9, and 11) to over 200 bp (exon 16). The signal peptide (MKFFIITCLLAVALA) is coded from the 13th to the 57th nt of exon 2, while the stop codon (TAA) is located at the 10th–12th nucleotide of exon 14. 

Interestingly, the AAACAGTTG duplication, which encodes the KQL tripeptide at the beginning of exon 13 as previously reported by Cosenza et al. [12], was not detected in any of the *CSN1S2* II mRNAs sequenced. Therefore, it is reasonable to assume that this duplication should be considered an artifact, similarly to what has been observed by Auzino et al. [7].

Compared to the *CSN1S2* I gene, the *CSN1S2* II gene has been less thoroughly investigated in donkeys and other species. Since its identification by Cosenza et al. [12] in Ragusana breed donkeys, its characterization has recently been carried out by Auzino et al. [7] only in Amiatina breed donkeys.

When comparing the results of the present work with those reported by these authors, significant differences in the variability of alternative splicing are evident. In particular, in all the *CSN1S2* II cDNA sequences analysed in the study by Auzino et al. [7], exons 3 and 7′ are constitutively missing and present, respectively (Table 2).

In contrast, none of the mRNA populations we observed are characterized by the skipping of exon 3, which encodes the ^3^EIKHVSSSE^11^ peptide, while the skipping of exon 7′, which encodes the KIELTKEEKLYLKQL (p.Asp50_Glu51ins15), is observed in only two of the detected transcript populations (Table 2). As reported by Auzino et al. [7], this exon appears to be nearly perfectly duplicated as exon 12, which encodes the peptide ^85^EIELSDEEKNYLKQL^99^ (Supplemental Figure S5). This exon is also involved in alternative skipping, further confirming that the phenomenon of coding sequences duplications observed in *CSN1S2* I is also present in *CSN1S2* II.

Alternatively, the sequence of the exon 7′ could be considered an “exonification” of an intronic region following the recognition of splice acceptor and donor sites. It is interesting to note that this intron has a triplet structure (45 bp), and its insertion does not alter the original reading frame, so the same primary amino acid sequence upstream and downstream of the insertion is maintained.

Alternative skipping observed in the present study, but not reported by Auzino et al. [7], involves exon 10 (encoding the KTSKKTVDM peptide) and exon 15 (105 bp) (Supplemental Figure S6). The latter is a non-coding exon (3′ UTR) and is therefore not responsible for variations in translation. Similarly to what was observed for exon 7′ at this locus or for exon 12′ at the *CSN1S2* I locus, it is likely that an intronic region is recognized as an exon during the splicing process of the primary transcript. Since the sequence of exon 15 and its splicing sites are perfectly conserved in *CSN1S2* II across *Equus asinus*, *caballus*, *quagga*, and *przewalskii*, it is very likely that a proportion of the mRNAs in these species may also contain this exon (Supplemental Figure S6).

It is plausible to hypothesize that the presence/absence of exon 15 could influence mRNA stability. Different studies have shown that 3′ UTRs are highly polymorphic in length and that a single gene can express multiple 3′ UTRs that differ in length, sequence, and assembly of regulatory motifs. It is known that the 3′ UTRs contain cis-regulatory elements recognized by trans-acting factors, and thus, changes in 3′ UTR length could alter several regulatory elements. In fact, many genes have an alternative 3′ UTR or contain internal introns that can switch 3′ UTR length. Therefore, changes in 3′ UTR length could affect gene expression by fine-tuning and reprogramming the mRNA regulatory landscape in cells [27,28].

In addition, similarly to what has been observed at exon 17 of *CSN1S2* I, exon 13 of the *CSN1S2* II gene is subject to the use of in-frame cryptic splice sites, leading to an alternative skipping of the first 33 nucleotides of exon 13 (GTGAAAATCAACCCAAAGTTCCCCTCTCCCCAG), which encodes the peptide ^100^VKINPKFPSPQ^110^ (Figure 2). We have never observed the alternative skipping of the first 33 nucleotides of exon 13 alone; it always occurs in conjunction with the simultaneous skipping of exons 11, 12, and/or 15, similar to what was reported by Auzino et al. [7] for the Amiatina breed (Table 2). A comparison of the sequences deposited in GeneBank shows that the presence of the cryptic splice sites at the 5′ of exon 13 is conserved among the different equid species (Supplemental Figure S7).

Concerning this event, the elimination of three nucleotides or those consisting of multiples of three from the mRNA, due to the recognition of a cryptic site of splice as an alternative to the canonical site (AG), is a feature common to the other casein-encoding genes. In particular, this event appears to be quite frequent in αs1-CN transcripts across various species, including sheep [29], cattle and buffalo [30], horse [31], goat [25], and humans [32].

Multiple forms of transcripts resulting from alternative splicing have also been demonstrated for αs2-CN in sheep [33,34], goats [35], buffalo [36,37], cattle [38], camels [21,23], and horses [39,40]. Similar defects in the processing of primary transcripts also characterize the αs1-CN gene (*CSN1S1*) in the same species [7,25,29,30,31,34,41,42,43,44,45], as well as in humans and pigs [33,46].

It has been proposed that exon skipping should be considered a frequent event when the coding region is divided into many short exons, as the maturation of long cognate primary transcripts appears to be an intricate process requiring many successive steps [34,47].

In general, alternative splicing resulting from the incorrect identification of donor or acceptor sites, leading to a shorter protein isoform compared to the full-length form or one characterised by premature stop codons, is a common event. This event has also been reported in other genes, such as *DGAT* 1 in cattle, yak, and buffalo [47].

### 3.2. Analysis of Genetic Diversity

#### 3.2.1. *CSN1S2* I and *CSN1S2* II cDNA Polymorphisms Detection

The analysis and alignment of the cDNA sequences of the eight subjects used in this study have highlighted a remarkable genetic diversity at both loci investigated: *CSN1S2* I and II.

At the *CSN1S2* I locus, the comparison of the obtained sequence evidenced seven SNPs, comprising six transitions and one transversion, five of which were non-synonymous SNPs. In particular, four of these polymorphisms are located in exons 8, 10, 14, and 17, respectively, whereas the remaining ones are located in exon 12 (Table S2). Two of these polymorphisms s (a transition T>C at the 119th nucleotide of exon 12, responsible for the amino acid changes p.I146>T, and a silent G>A transition at the 12th nucleotide of exon 14) have already been reported by Cosenza et al. [12]. 

Similarly, the *CSN1S2* II locus also seems to be characterized by notable polymorphisms. The comparison of the cDNA sequences revealed nine SNPs (eight transitions and one transversion), six of which resulted in putative amino acid exchange: p.L63>F, p.H70>Q, p.D90>N, p.A129>T, p.H131>Y, and p.E144>G (Table S3).

The non-synonymous SNP at the nucleotide 43 of exon 13 (p.A129>T) has already been observed by Auzino et al. [7], whereas the presence of the G in position 86 of exon 13, as reported in FN298386, was not observed in any of the analysed transcripts (Table S3).

The presence of such a high level of polymorphisms at the *CSN1S2* locus is not unusual. In fact, studies of αs2-CN have revealed diverse protein and DNA polymorphisms across different species. In horses, a total of six non-synonymous single-nucleotide variants and one large deletion leading to eight distinct putative αs2-CN isoforms were identified [48]. Among ruminants, goats and sheep exhibit a higher level of genetic diversity at this locus, with at least eight and seven alleles characterized in the two species, respectively [26,35,49]. Similarly, eight alleles have been identified in buffalo, three of which are deleted alleles. These deleted alleles are characterized by the alteration of the acceptor splice site of exon 7, which leads to the skipping of this exon at the mRNA level [36]. Conversely, in cattle, only four variants have been characterized [50]. 

Recently, the characterization of the *CSN1S2* gene has revealed genetic variations also in the Old-World Camels [21]. Such a high level of polymorphism is comparable only to those observed in the *CSN1S1*, *CSN2*, and *CSN3* genes in goats [51,52] or *CSN3* gene in cattle [53].

SNP detection through mRNA sequencing is particularly valuable for livestock species, as whole genome sequencing is expensive and exome sequencing tools are not available. Furthermore, SNPs detected in expressed regions can be useful for characterizing variants that affect protein function. However, data derived from RNA analyses are often considered a less-than-ideal source for SNP detection due to a higher false positive rate, which arises from both biological and technical factors. For example, RNA polymerases are known to commit errors through a process termed transcriptional mutagenesis. This process affects both mitotic and post-mitotic cells, as it is independent of DNA replication. Consequently, errors in transcription are generally considered transient, with no long-term consequences, and are not stably inherited from cell to cell. However, several lines of evidence now demonstrate that transcription errors are not always random or temporary, and that they can have lasting consequences, particularly in human health [54]. Technical factors contributing to errors include reverse transcription errors, which occur at a certain rate during the conversion of RNA to cDNA and are a mandatory step for RNA cloning, PCR amplification errors, and the error-prone nature of sequencing itself [55].

Therefore, to confirm the presence of the SNPs at donkey *CSN1S2* I and II, uniquely identified through RNA cloning and sequencing, and to determinate their frequency, a future objective will be to sequence the exonic regions at DNA level or to carry out genotyping for these markers on a suitable number of donkeys belonging to the Ragusana breed. 

#### 3.2.2. DNA Sequences: Detection of a Point Mutation in the Splice Acceptor Site of *CSN1S2* I Exon 17

As previously described, the screening of clones for the Ragusana *CSN1S2* I gene and the subsequent sequencing revealed transcripts characterized by the alternative skipping of the first 15 nucleotides of exon 17. This results in an αs2-CN I isoform missing the pentapeptide ^176^NKINQ^180^. This event has been previously reported in the same breed by Saletti et al. [17] and Cunsolo et al. [16]. Conversely, Auzino et al. [7] did not observe any αs2-CN I isoforms lacking the pentapeptide ^176^NKINQ^180^ in the Amiatina breed.

It has therefore been hypothesized that the Ragusana breed, or more generally the donkey species, may be characterized by a polymorphism affecting the splice acceptor site of *CSN1S2* I exon 17. In order to demonstrate this hypothesis, we sequenced exon 17 and flanking introns of the *CSN1S2* I gene of eight Ragusana and eight Amiatina donkeys. The sequence comparison confirmed a point mutation (FM946022.1: c.375-1G>A) at the splice acceptor site of exon 17 in the *Equus asinus CSN1S2* I gene in both breeds, resulting in allele-specific partial exon skipping (Supplemental Figure S8). 

It is therefore possible to hypothesize that in donkeys homozygous for guanine (c.375-1G) the canonical splice site of exon 17 (long form) is recognized, while in donkeys homozygous for the adenine (c.375-1A) it would translate an αs2-CN isoform constitutively deleted of the ^176^NKINQ^180^ peptide (short form) similar to what has been reported for equine species.

**Figure 3:**
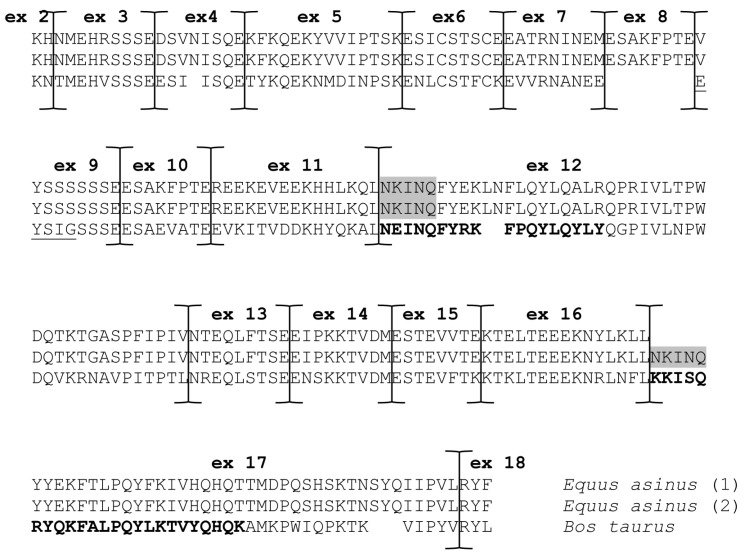
Comparison of deduced *Equus asinus* (present work) and *Bos taurus* (GenBank: AAI14774.1) amino acid sequences of mature αs2-casein. Major bovine IgE-binding regions (corresponding amino acid sequences: 83–100 and 165–188) are in bold. Donkey sequences ^92^NKINQ^96^ and ^176^NKINQ^180^ are shaded. Alignment was performed using DNAsis pro Software v2.0 (Hitachi).

It is interesting to note that the amino acid sequences NKINQ are traits of some IgE-binding epitopes of bovine αs2-CN. In particular, the NKINQ sequence is a trait of two major IgE-binding epitopes of bovine αs2-CN (Figure 3) and therefore could be related to the already demonstrated low allergenic properties of donkey’s milk [17].

The alteration of a splice acceptor/donor site, along with the resulting exon skipping, is an unusual event, although it has been observed in other species at this locus, such as in buffalo [36,37], or at other loci, like *CSN1S1* in cattle [56] and sheep [43]. Therefore, similar to these examples, this case represents an intra-species allele-specific event, rather than a constitutive condition as reported for horses [17].

#### 3.2.3. Genotyping of the SNP FM946022.1: c.375-1G>A in the Donkey *CSN1S2* I Gene

**Figure 4:**
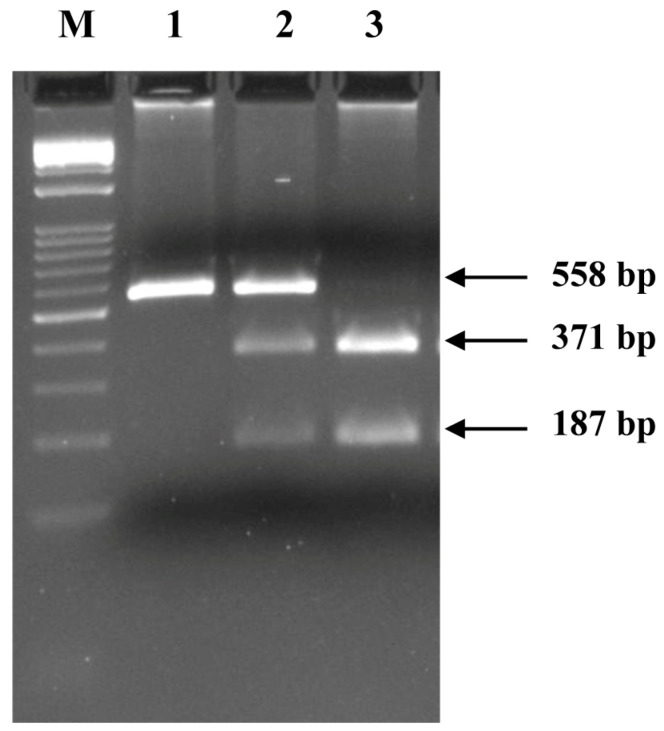
Observed genotypes after *Xba*I (T/CTAGA) digestion of PCR-amplified fragments spanning exon 17 and its flanking regions in donkeys carrying the FM946022.1: c.375-1G>A mutation in the splice acceptor site of exon 17 in the *CSN1S2* I gene. Lane 1, AA homozygous sample; lane 2, heterozygous sample; lane 3, GG homozygous sample. The marker (M) used is the 1kb Opti-DNA Ladder (0.1–10 kb) (Applied Biological Materials, ABM, Vancouver, BC, Canada).

The c.375-1G>A transition at the acceptor splice site of exon 17 of the *CSN1S2* I gene alters a restriction site for the endonuclease *Xba*I (T/CTAGA). Consequently, a PCR-RFLP protocol was set up for the quick genotyping of samples. Digestion of the PCR product (558 bp) allows for the identification of both alleles. The restriction pattern is characterized by one undigested fragment in c.375-1A homozygous samples, whereas the same amplicon is restricted into two fragments of 187 and 371 bp in samples homozygous for c.375-1G. The restriction pattern of heterozygous samples shows all three fragments (Figure 4).

The genotype distributions and allelic frequencies of the polymorphism, determined in all 119 investigated donkeys (105 Ragusana and 14 Amiatina), are reported in Table S4.

Across the total investigated population, the G allele had a frequency of 0.7563, and the χ2 value (0.2818) indicated no significant deviation from the Hardy–Weinberg equilibrium (*p* ≤ 0.05). 

It should be noted that among the 14 Amiatina donkeys, only two genotypes were observed: G/G (*n* = 9) and A/G (*n* = 5). Although the number of subjects investigated is limited, the allele and genotype frequencies are comparable to those observed in the Ragusana breed. Therefore, it is conceivable that the Amiatina donkeys studied by Auzino et al. [7] were homozygous for the c.375-1G allele, which could explain the absence of transcripts characterized by the skipping of the first 15 nucleotides of exon 17.

From the comparison of the sequences deposited in GeneBank, it is interesting to note that the presence of adenine, responsible for the skipping of the first 15 nt of the exon 17 at mRNA level, although present at low frequency in the donkey species (0.2437), appears to be a constitutive condition in all other species belonging to the different families within the *Perissodactyla* order: *Equidae* (horses and zebras) *Rhinocerotidae* (rhinoceroses), and *Tapiridae* (tapirs) (Supplemental Figure S9). Furthermore, all these perissodactyl species are characterized by the presence of a second splice acceptor site, which enables recognition of the exon 17 deletion of the first 15 nucleotides during the splicing mechanism (Supplemental Figure S9).

Among all the species belonging to the orders *Cetartiodactyla* or *Carnivora*, the simultaneous presence of both the canonical and cryptic acceptor sites of the 17th exon is observed only in those within the sub-order *Ruminantia* (Supplemental Figure S9). However, to date, no studies have reported a possible alternative skipping of the first 15 nucleotides of exon 17 at the *CSN1S2* mRNA level in these species.

In general, it is possible that the G allele represents the ancestral condition of the *CSN1S2* gene, as it is present in all species within the orders *Cetartiodactyla* and *Carnivora* for which sequences are available in GeneBank (Supplemental Figure S9). During the evolution of *Perissodactyla*, a G>A transition may have occurred, leading to the fixation of adenine in all families of this order, with the exception of the donkey species. Investigations are currently underway to assess whether this locus is also polymorphic in the remaining perissodactyl species, which could indicate the presence of a Trans-Specific Polymorphism (TSP), similar to what has been reported for other *Cetartiodactyla* species, such as hormone-encoding genes in goats, sheep, and buffalo [57,58] or milk protein-encoding genes in camelids [59]. TSPs are ancient genetic variants whose origin predates speciation events, resulting in shared alleles among evolutionarily related species [60]. Therefore, considering these observations, the G>A transition at the splice acceptor site of exon 17, in addition to being responsible for modifying the splicing mechanism and, consequently, associated with the regulation of donkey *CSN1S2* I gene expression, could serve as a valuable marker for inter- and intra-species phylogenetic analysis.

The question of the phylogenetic origin of species is indeed complex and is often debated. In particular, the evolutionary dynamics between the orders *Cetartiodactyla* and *Perissodactyla* are somewhat complicated. Most studies suggest that these two ungulate groups form a clade (*Euungulata*), while the groups *Carnivora* and *Philidota* form the clade *Ferea*. Furthermore, in many phylogenies, *Ferea* and *Euungulata* are grouped together within a larger clade called *Ferungulata* [61]. However, the issue remains a subject of ongoing debates.

## 4. Conclusions

This study provides a comprehensive analysis of the transcripts and genetic variability within the *CSN1S2* I and II loci in the domestic Ragusana breed of donkeys. We have elucidated the genetic events responsible for the various transcripts and described alternative splicing events that contribute to the complex expression patterns of donkey αs2-CNs. However, it is also reasonable to hypothesize the existence of transcripts forms others than those reported in this study, which have not been detected so far due to their existing in undetectable amounts. Significant genetic diversity at both the loci has been found. One example is the FM946022.1: c.375-1G>A transition at the splice acceptor site of exon 17 that results in allele-specific partial exon skipping. The presence of A seems to be a constitutive feature of the other perissodactyl species, indicating a possible evolutionary shift from the ancestral G allele common in Cetartiodactyla and Carnivora. Further investigation is needed to explore the potential trans-specific nature of this polymorphism across different species and its implications for gene expression regulation. Finally, our findings underscore the importance of continued study of the genetic diversity of donkey breeds, both for species conservation and for improving the quality and safety of donkey milk for human consumption. 

## Figures and Tables

**Table 1:** Ragusana donkey *CSN1S2* I mRNAs identified through clone screening and comparison with donkey *CSN1S2* I transcripts characterized in previous studies.

	Present Work	Auzino et al. [7]	Saletti et al. [17] Cunsolo et al. [16]	Deduced Mature Protein Size (aa)	Theoretical Mw
	Correctly assembled	-	Correctly assembled	221	26,030.19
Alternative skipping	Lacking exon 11	-	-	206	24,116.04
	Insertion exon 12′	-	-	143	16,617.68
	-	Lacking exon 3 and 3′ end of exon 17	-	205	24,145.24
	Lacking exons 4, 5, 6, and 3′ end of exon 17	Lacking exons 4, 5, 6, and 3′ end of exon 17	Lacking exons 4, 5, 6, and 3′ end of exon 17	183	21,713.42
	Lacking 3′ end of exon 17	Lacking 3′ end of exon 17	Lacking 3′ end of exon 17	214	25,203.33
	Lacking exons 4, 5, and 6	Lacking exons 4, 5, and 6	-	190	22,540.28
	-	Lacking exons 4, 5, 6, and 13	-	181	21,490.19
	-	Lacking exon 6 and 3′ end of exon 17	-	205	24,263.32
	Lacking exons 4, 5, 6, and 5′ of exon 17	-	Lacking exons 4, 5, 6, and 5′ of exon 17	185	21,942.60
	Lacking 5′ of exon 17	-	Lacking 5′ of exon 17	216	25,432.52

**Table 2:** Ragusana donkey *CSN1S2* II mRNAs identified through clone screening and comparison with Amiatina donkey *CSN1S2* II transcripts characterized by Auzino et al. [7].

	Present Work	Auzino et al. [7]	Deduced Mature Protein Size (aa)	Theoretical Mw
	Correctly assembled with or without exon 15		142	16,377.88
Alternative skipping	Insertion exon 7′		157	18,236.13
	Lacking exon 9 and insertion exon 7′		149	17,314.17
		Lacking exon 3 and insertion exon 7′	148	17,239.06
		Lacking exons 3, 9, and insertion exon 7′	140	16,317.10
		Lacking exons 3, 11, 12, first 33nt exon 13, and insertion exon 7′	114	13,308.63
	Lacking exon 9		134	15,455.92
	Lacking exon 10		133	15,358.65
	Lacking exons 11, 12, and first 33nt exon 13 with or without exon 15		108	12,447.45
	Lacking exons 9, 10, and 15		125	14,436.69

## Data Availability

The data presented in this study are available on request from the corresponding author.

## Supplementary Materials

The following supporting information can be downloaded at: https://www.mdpi.com/article/10.3390/ani14202918/s1. Figure S1: Results of *CSN1S2* I cDNA sequencing. Alternative skipping of the first 15 nucleotides of exon 17 (AACAAAATCAACCAG). The large arrows indicate the exons; Figure S2: Comparison of *Equus asinus* genomic sequences covering (**A**) exons 11 and 16 and the flanking regions of the *CSN1S2* I gene (GeneBank JADWZW020000003.1 from 152928702 to 152928763 and from 152932720 to 152932781); (**B**) exons 12 and 17 and the flanking regions of the *CSN1S2* I gene (GeneBank JADWZW020000003.1 from 152930146 to 152930310 and from 152933634 to 152933793); (**C**) exons 8 and 10 and the flanking regions of the *CSN1S2* I gene (GeneBank JADWZW020000003.1 from 152928328 to 152928636 and from 152926989 to 152927297). Exon sequences are in uppercase and bold letters. Acceptor and donor splice sites are underlined. Dashes represent identical nucleotides in the upper lines. Conserved amino acids are shaded. Alignment was performed using DNAsis pro Software v2.0 (Hitachi); Figure S3: (**A**) Results of the *CSN1S2* I cDNA sequencing. Alternative skipping of the last 35 nucleotides of exon 17. The large arrows indicate exons. (**B**) Comparison of *Camelus dromedarius* (GeneBank OQ730239 from 12122 to 12280) and *Equus asinus* (GeneBank JADWZW020000003.1 from 152933627 to 152933786) genomic sequences covering exons 15 and 17 of the *CSN1S2* I gene, along with the respective flanking regions. Exon sequences and amino acids are in uppercase and bold letters. Acceptor and donor splice sites are underlined and shaded. Dashes represent identical nucleotides to those in the upper lines. Conserved amino acids are also shaded. Alignment was performed using DNAsis pro Software v2.0 (Hitachi); Figure S4: (**A**) Comparison of *Equus asinus* (GeneBank JADWZW020000003.1 from 152930069 to 152930978), *Equus quagga* (GeneBank JAKJSB010001568.1 from 100897185 to 100896302, complement), *Equus caballus* (GeneBank PJAA01000004.1 from 66588578 to 66587670), and *Equus przewalskii* (GeneBank ATBW01083582.1 from 4801 to 3893, complement) genomic sequences covering exons 12 to 13 of the *CSN1S2* I gene and their flanking regions. Dashes represent identical nucleotides to those in the upper lines. Exon sequences and amino acids are in uppercase and bold letters, and an asterisk indicates the premature termination stop codon. Acceptor and donor splice sites are underlined and shaded. Alignment was performed using DNAsis pro Software v2.0 (Hitachi). (**B**) Results of the *CSN1S2* I cDNA sequencing. The large arrows indicate exons; Figure S5: (**A**) Comparison of *Equus asinus* exon 7′ and 12 sequences of the *CSN1S2* II gene. Dashes represent identical nucleotides to those in the upper lines. Conserved amino acids are shaded. Alignment was performed using DNAsis pro Software v2.0 (Hitachi). Results of *CSN1S2* II cDNA sequencing without (**B**) and with (**C**) exon 7′ (in red) sequence. The large arrows indicate exons; Figure S6: Results of *CSN1S2* II cDNA sequencing. (**A**) Alternative skipping of exon 10. (**B**) Alternative skipping of exon 15; the large arrows indicate exons. (**C**): Comparison of *Equus asinus* (GeneBank PSZQ01005937.1 from 24169059 to 24169178, complement), *Equus quagga* (GeneBank JAKJSB010001568.1 from 100955081 to 100954962, complement), *Equus caballus* (GeneBank PJAA01000004.1 from 66663354 to 66663473, complement), and *Equus przewalskii* (GeneBank ATBW01081756.1 from 33803 to 33922, complement) genomic sequences covering exon 15 and flanking regions of the *CSN1S2* II gene. Exon sequences are in uppercase and bold letters. Acceptor and donor splice sites are underlined and shaded. Dashes represent identical nucleotides to those in the upper lines. Alignment was performed using DNAsis pro Software v2.0 (Hitachi); Figure S7: (**A**) Results of *CSN1S2* II cDNA sequencing. Alternative skipping of exons 11 and 12 and of the first 33 nucleotides of exon 13. The large arrows indicate exons. (**B**) Comparison of *Equus asinus* (GeneBank PSZQ01005937.1 from 24166892 to 24167071), *Equus caballus* (GeneBank PJAA01000004.1 from 66661191 to 66661370), *Equus quagga* (GeneBank JAKJSB010001568.1 from 100957247 to 100957068, complement), and *Equus przewalskii* (GeneBank ATBW01081756.1 from 31501 to 31680) genomic sequences covering exon 13 and flanking regions of the *CSN1S2* II gene. Exon sequences and amino acids are in uppercase and bold letters. Canonical acceptor and donor splice sites and cryptic acceptor sites are underlined and shaded. Dashes represent identical nucleotides to those in the upper lines. Alignment was performed using DNAsis pro Software v2.0 (Hitachi); Figure S8: Results of the DNA sequencing of exon 17 and flanking regions of the donkey *CSN1S2* I gene. (**A**) A/A homozygous sample. (**B**) G/G homozygous sample. (**C**) heterozygous sample. The acceptor splice sites of exon 17 are underlined. Solid arrows indicate the SNP (transition FM946022.1:c.375-1G>A) located in the acceptor splice site; Figure S9: Comparison of genomic sequences covering exon 17 of the donkey *CSN1S2* I gene and predicted amino acid sequences with corresponding sequences from representative species of the *Perissodactyla*, *Cetartiodactyla*, and *Carnivora* orders. Exon sequences are in uppercase and bold letters. Amino acid sequences are highlighted. Canonical and cryptic acceptor and donor splice sites are shaded. Alignment was performed using DNAsis pro Software v2.0 (Hitachi); Table S1: Oligonucleotide primers used for the characterization of the donkey *CSN1S2* I and *CSN1S2* II cDNAs; Table S2: Detection of polymorphisms at *CSN1S2* I locus in Ragusana donkeys and corresponding electropherograms; Table S3: Polymorphisms detected at *CSN1S2* II cDNA in Ragusana donkeys, corresponding electropherograms, and comparison with counterpart sequences available in the literature and GeneBank; Table S4: Genotyping data and allele frequency of the FM946022.1:c.375-1G>A transition at the acceptor splice site of exon 17 in the *CSN1S2* I gene in Ragusana and Amiatina donkeys.
