# Supplementary material for: A Comprehensive Analysis of CSN1S2 I and II Transcripts Reveals Significant Genetic Diversity and Allele-Specific Exon Skipping in Ragusana and Amiatina Donkeys
Source: Animals (Basel). 2024 Oct 10;14(20):2918. doi: 10.3390/ani14202918 (PMC11503821; doi:10.3390/ani14202918)
Supplement: Supplementary file 1 [file animals-14-02918-s001.zip › Figure S1.pdf]

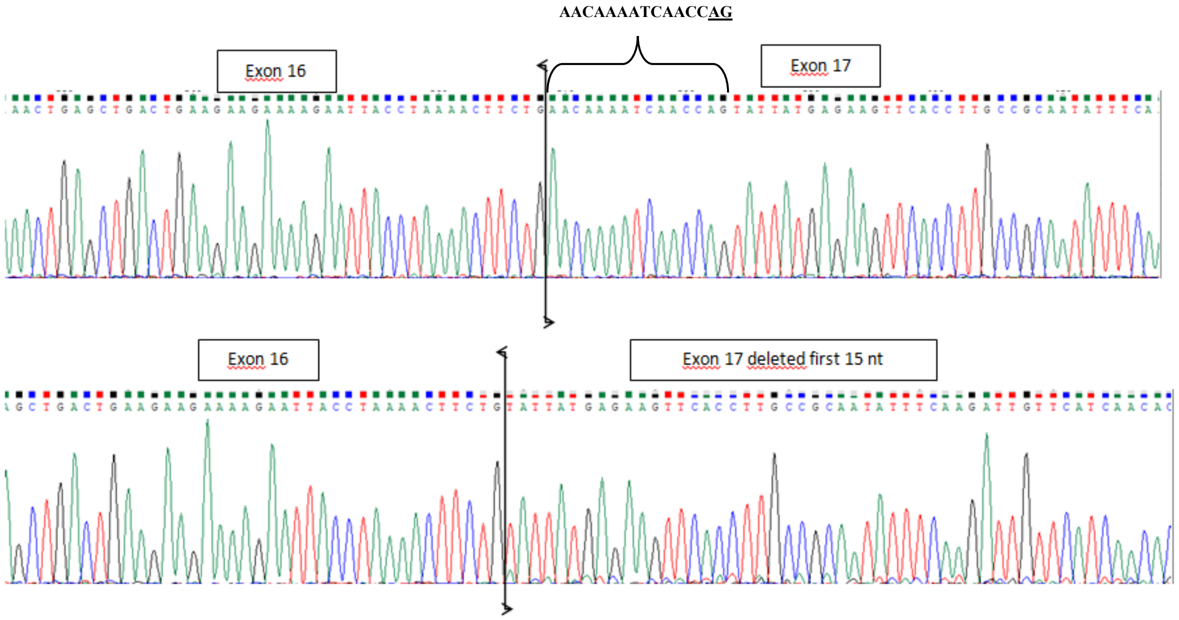

Figure S1. Results of *CSN1S2* I cDNA sequencing. Alternative skipping of the first 15 nucleotides of exon 17 (AACAAAATCAACCAG). The in-frame cryptic splicing acceptor site is underlined. The large arrows indicate the exons.
