# Supplementary material for: A Comprehensive Analysis of CSN1S2 I and II Transcripts Reveals Significant Genetic Diversity and Allele-Specific Exon Skipping in Ragusana and Amiatina Donkeys
Source: Animals (Basel). 2024 Oct 10;14(20):2918. doi: 10.3390/ani14202918 (PMC11503821; doi:10.3390/ani14202918)
Supplement: Supplementary file 1 [file animals-14-02918-s001.zip › Figure S2.pdf]

```
 R  E  E  K  E  V  E  E  K  H  H  L  K  Q  L
AGAGAAGAAAAGGAAGTGGAAGAAAAGCACCACCTAAAACAACTGgtaaacttctcatacaa      Exon 11
-A-ACT--GCT-ACT-AA---------A-TT---------TT--------t--tc----g--      Exon 16
 K  T  E  L  T  E  E  E  K  N  Y  L  K  L  L
```

A

```
                    N  K  I  N  Q  F  Y  E  K  L  N  F  L  Q  Y  L  Q  A  L  R  Q
ataatttcttttctttctagAACAAAATCAACCAGTTTTATGAGAAGTTGAACTTCCTCCAATATCTCCAGGCCCTTCGTCAA
-------t---     -------------------A-----------C-C---G-CG------T--A--ATTG---A----
                    N  K  I  N  Q  Y  Y  E  K  F  T  L  P  Q  Y  F  K  I  V  H  Q
```

B

```
 P  R  I  V  L  T  P  W  D  Q  T  K  T  G  A  S  P  F  I  P  I  V
CCTCGGATTGTCCTGACCCCGTGGGATCAGACTAAGACAGGGGCCTCCCCCTTTATTCCTATTGTGgtgagcgctgcttttt   Exon 12
-AC-A--CAACTA--GAT--ACA-AG---C-G-------AATT-T-A--AAA----C--CG--C-------tt--c------   Exon 17
 H  Q  T  T  M  D  P  Q  S  H  S  K  T  N  S  Y  Q  I  I  P  V  L
```

```
atttgatattgtacaaataatgcagaattgatgaaaattttgaaatgaaacattttgagt
----------------------t-------------------------------------

catgttattactttgacacagaatgagggctctgatgataaatattttaatttaatgaat
-g------------t---------------------------------------------

tcctaataaatgatataatttctgaggagtagtggcataggccccaaacattcaattatt
------------------------------------------------------------

tgaccctatttatgtagtacaactttaaggctaaatcatgcaagttaagttaactctcct
------------------------------------------------------------
               E  S  A  K  F  P  T  E
tttcttcccataagGAATCTGCTAAATTTCCCACAGAGgtaagtaattttaattcacata      Exon 8
--------------GAATCTGCTAAATTTCCCACAGAG----------------------      Exon 10

aaatgaaat
---------
```

C

Figure S2. Comparison of *Equus asinus* genomic sequences covering (**A**) exons 11 and 16 and the flanking regions of the *CSN1S2* I gene (GeneBank JADWZW020000003.1 from 152928702 to 152928763 and from 152932720 to 152932781); (**B**) exons 12 and 17 and the flanking regions of the *CSN1S2* I gene (GeneBank JADWZW020000003.1 from 152930146 to 152930310 and from 152933634 to 152933793); (**C**) exons 8 and 10 and the flanking regions of the *CSN1S2* I gene (GeneBank JADWZW020000003.1 from 152928328 to 152928636 and from 152926989 to 152927297). Exon sequences are in uppercase and bold letters. Acceptor and donor splice sites are underlined. Dashes represent identical nucleotides in the upper lines. Conserved amino acids are shaded. Alignment was performed using DNAsis pro Software v2.0 (Hitachi)
