# Supplementary material for: A Comprehensive Analysis of CSN1S2 I and II Transcripts Reveals Significant Genetic Diversity and Allele-Specific Exon Skipping in Ragusana and Amiatina Donkeys
Source: Animals (Basel). 2024 Oct 10;14(20):2918. doi: 10.3390/ani14202918 (PMC11503821; doi:10.3390/ani14202918)
Supplement: Supplementary file 1 [file animals-14-02918-s001.zip › Figure S3.pdf]

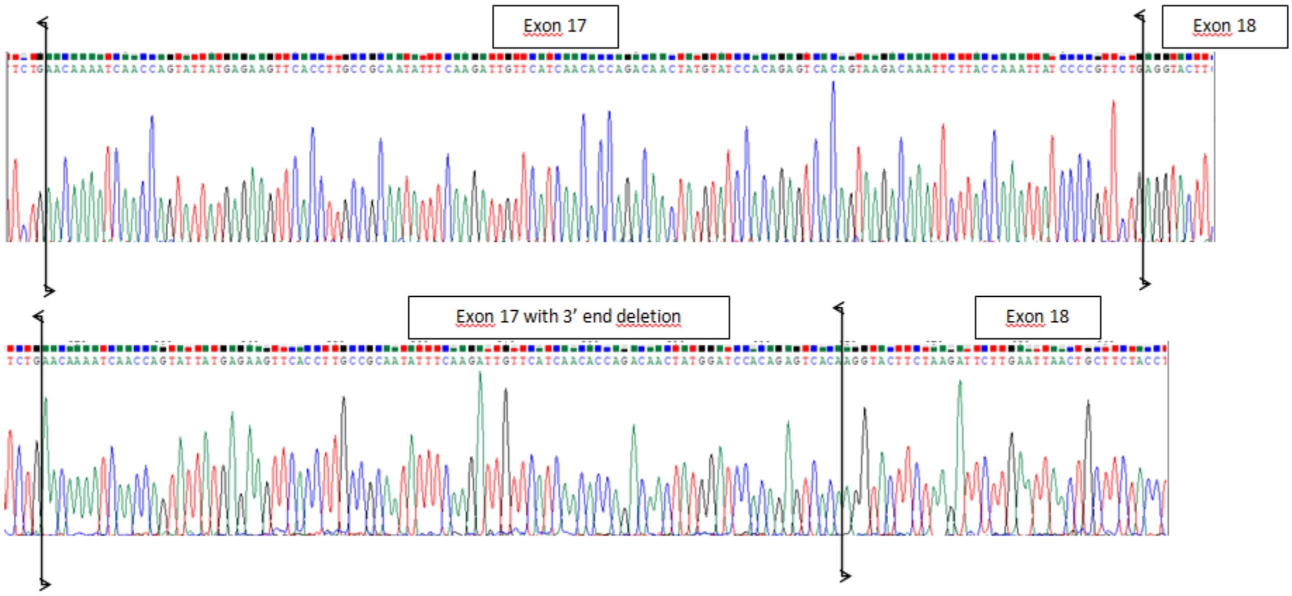

A

```
                                            N  K  I  Y  Q  Y  Y  Q  T  F  L  W  P  E  Y  L  K  T  V
attacttacaattttttttc agAACAAAATCTACCAATATTATCAGACATTCCTCTGGCCAGAGTATCTCAAGACTGTTT
-----c--t----------t-----------A----G------G----G---AC--T---GC-A---T------T----C
                                            N  K  I  N  Q  Y  Y  E  K  F  T  L  P  Q  Y  F  K  I  V
```

B

```
Y  Q  Y  Q  K  T  M  T  P  W  N  H  I  K  V  K  A  Y  Q  I  I  P  N  L
ATCAATATCAGAAAACTATGACTCCATGGAATCACATCAAGGTAAAAGCTTACCAAATTATTCCCAATTTGgtgagttct
-----C-C----C-------GA----CA--G-----GT---AC---TT-------------C---GT-C-----------
H  Q  H  Q  T  T  M  D  P  Q  S  H  S  K  T  N  S  Y  Q  I  I  P  V  L
```

```
accttttatttgtagtttta   Exon 15   Camelus dromedarius
c------tac-t-gaa----   Exon 17   Equus asinus
```

Figure S3. (**A**) Results of the *CSN1S2* I cDNA sequencing. Alternative skipping of the last 35 nucleotides of exon 17. The large arrows indicate exons. (**B**) Comparison of *Camelus dromedarius* (GeneBank OQ730239 from 12122 to 12280) and *Equus asinus* (GeneBank JADWZW020000003.1 from 152933627 to 152933786) genomic sequences covering exons 15 and 17 of the *CSN1S2* I gene, along with the respective flanking regions. Exon sequences and amino acids are in uppercase and bold letters. Acceptor and donor splice sites are underlined and shaded. Dashes represent identical nucleotides to those in the upper lines. Conserved amino acids are also shaded. Alignment was performed using DNAsis pro Software v2.0 (Hitachi).
