# Supplementary material for: A Comprehensive Analysis of CSN1S2 I and II Transcripts Reveals Significant Genetic Diversity and Allele-Specific Exon Skipping in Ragusana and Amiatina Donkeys
Source: Animals (Basel). 2024 Oct 10;14(20):2918. doi: 10.3390/ani14202918 (PMC11503821; doi:10.3390/ani14202918)
Supplement: Supplementary file 1 [file animals-14-02918-s001.zip › Figure S4.pdf]

```
aaataaagttctaagtaatttagctctccaagatcttcttgccccatggaggagattcaa
-------------------------------------------- ---------------
--------------------------------------------- --------------
--------------------------------------------- --------------

                                      N  K  I  N  Q  F  Y  E
tgttggagcaaagattgataatttcttttctttctagAACAAAATCAACCAGTTTTATGA
------------------------------------------------------------
------------------------------------------------------------
------------------------------------------------------------

  K  L  N  F  L  Q  Y  L  Q  A  L  R  Q  P  R  I  V  L  T  P
GAAGTTGAACTTCCTCCAATATCTCCAGGCCCTTCGTCAACCTCGGATTGTCCTGACCCC
---------------------------------------------------T--------   Exon 12
------------------------------------------------------------
--------------------------------------------A---------------
                                            Q

  W  D  Q  T  K  T  G  A  S  P  F  I  P  I  V
GTGGGATCAGACTAAGACAGGGGCCTCCCCCTTTATTCCTATTGTGgtgagcgctgcttt
A-----------------------------------------------------------
-----------------------A------------------------------------
                       D
-----------------------A------------------------------------
                       D

ttgtcgtttgtggttttgcttttggttctgtttttcttctttgtttttggggagggatga
------------------------t-----------------------------------
------------------------------------------------------------
----t-------------------------------------------------------

gctaaggataaagatatgtaaaatagctagagacaaaatgcccaacaagttctaactcag
------------------c---------------------t-------------------
------------------------------------------------------------
---------------------------------------------------------t--

aaagtagaacaattgtaaagaaaatcttatagtccagaaattaaatcataaacaatatag
------------------------------------------------------------
------------------------------------------------------------
------------------------------------------------------------

tccagatatagtaataaatgaaagtaacaataatgacaaataagtaatttcatttaaatt
---------------------------------c-----------------c--------
------------------------------------------------------------
---------------------------------c--------------------------

taacatatgtgtcctaaatgttgctcatatttatttgcttggttcttccaacaattct
------------------------------a----------------c------------
---------------------c--------------------------------------
------------------------------------------------------------

          E  G  I  E  I  I  I  F  M  *
ctaatgcagGAGGGGATAGAAATCATTATCTTCATGTAACGAAAAGACTGTAATGTAAGA      Exon 12'      A
------------------------------------------------------------
------------------------------------------------------------
------------------------------------------------------------

AAATGCAAGGAAGTGAAGTAACTCACACGACTTTCAAGTTCTTTCTTCCAGTTTgtttca
------------A--------------------C--------------------------
---------------------------------C--------------------------
---------------------------------C--------------------------

cgaaacagttttgtcttagggaaatttttcttatatgaggaataaataacaaaagtcctc
------------------------------------------------------------
------------------------------------------------------------
------------------------------------------------------------

                                     N  T  E  Q  L  F  T  S
ttgtcttggcctttttttttttttttctgggatacagAACACAGAACAGCTCTTCACCAGT      Exon 13
------------------------ -----------------------------------
------------------------------------------------------------
------------------------------------------------------------

 E
GAGgtaaggcatttactacaaacacaatatctcagtaactagcaagatattatacactt
------------------------------------------------------------
---------------------------g--------------------------------
---------------------------g--------------------------------
```

```
attatgcattgtactagagtctttgaactctttttctaagcaataattctcaatttagaa
---------------------------------------------------------c--
---------------------------------------------------------c--
---------------------------------------------------------c--

aatatggtag  Equus asinus
----------  Equus quagga
----------  Equus caballus
----------  Equus przewalskii
```

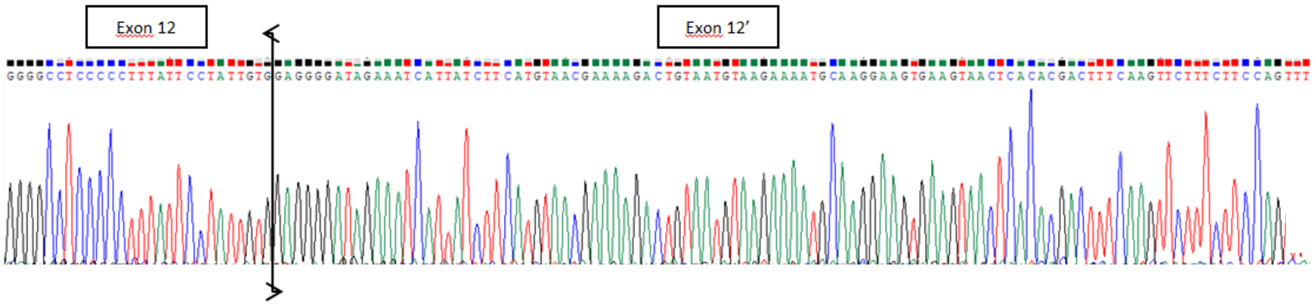

B

Figure S4. (**A**) Comparison of *Equus asinus* (GeneBank JADWZW020000003.1 from 152930069 to 152930978), *Equus quagga* (GeneBank JAKJSB010001568.1 from 100897185 to 100896302, complement), *Equus caballus* (GeneBank PJAA01000004.1 from 66588578 to 66587670), and *Equus przewalskii* (GeneBank ATBW01083582.1 from 4801 to 3893, complement) genomic sequences covering exons 12 to 13 of the *CSN1S2* I gene and their flanking regions. Dashes represent identical nucleotides to those in the upper lines. Exon sequences and amino acids are in uppercase and bold letters, and an asterisk indicates the premature termination stop codon. Acceptor and donor splice sites are underlined and shaded. Alignment was performed using DNAsis pro Software v2.0 (Hitachi). (**B**) Results of the *CSN1S2* I cDNA sequencing. The large arrows indicate exons.
