# Supplementary material for: A Comprehensive Analysis of CSN1S2 I and II Transcripts Reveals Significant Genetic Diversity and Allele-Specific Exon Skipping in Ragusana and Amiatina Donkeys
Source: Animals (Basel). 2024 Oct 10;14(20):2918. doi: 10.3390/ani14202918 (PMC11503821; doi:10.3390/ani14202918)
Supplement: Supplementary file 1 [file animals-14-02918-s001.zip › Figure S5.pdf]

A

```
 K  I  E  L  T  K  E  E  K  L  Y  L  K  Q  L
AAAATTGAGCTGACTAAGGAAGAGAAGCTCTACCTAAAACAGCTG   Exon 7′
G-----------G-G-T-----A---AAT-----G------T--   Exon 12
 E  I  E  L  S  D  E  E  K  N  Y  L  K  Q  L
```

B

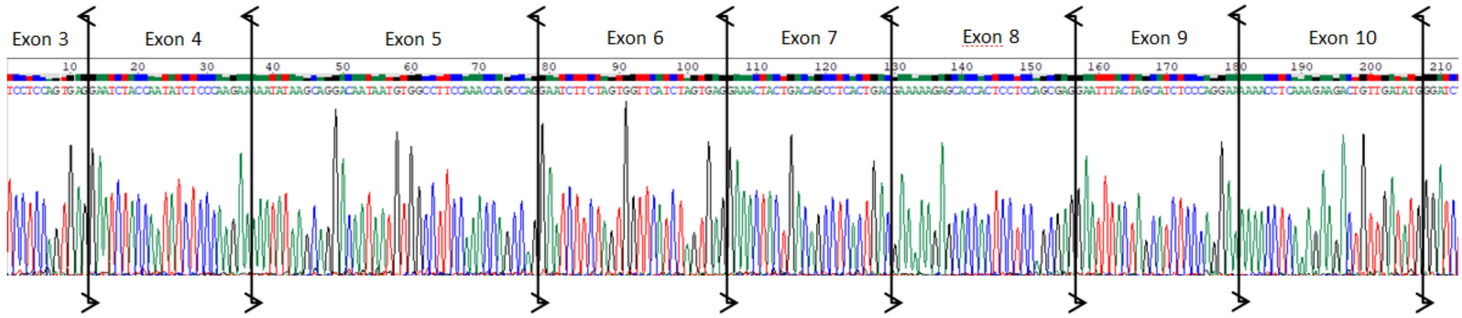

C

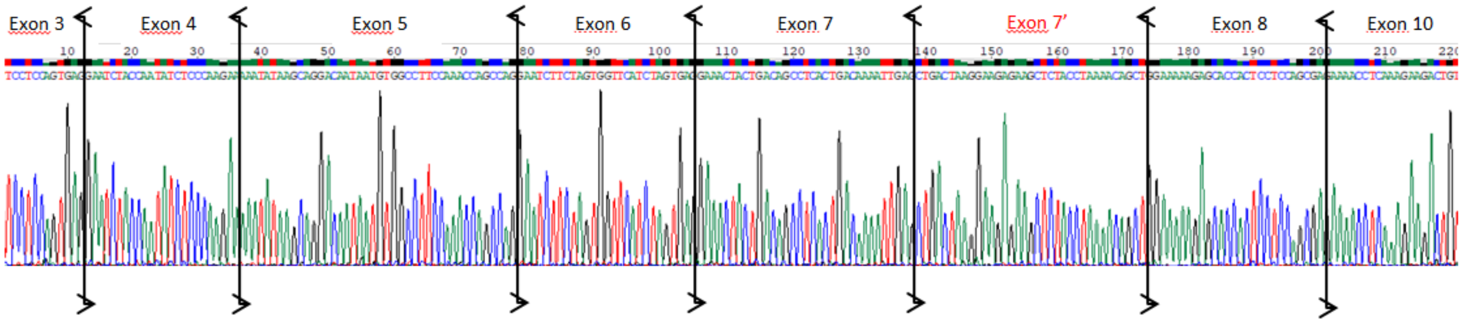

Figure S5. (**A**) Comparison of *Equus asinus* exon 7′ and 12 sequences of the *CSN1S2* II gene. Dashes represent identical nucleotides to those in the upper lines. Conserved amino acids are shaded. Alignment was performed using DNAsis pro Software v2.0 (Hitachi). Results of *CSN1S2* II cDNA sequencing without (**B**) and with (**C**) exon 7′ (in red) sequence. The large arrows indicate exons.
