# Supplementary material for: A Comprehensive Analysis of CSN1S2 I and II Transcripts Reveals Significant Genetic Diversity and Allele-Specific Exon Skipping in Ragusana and Amiatina Donkeys
Source: Animals (Basel). 2024 Oct 10;14(20):2918. doi: 10.3390/ani14202918 (PMC11503821; doi:10.3390/ani14202918)
Supplement: Supplementary file 1 [file animals-14-02918-s001.zip › Figure S6.pdf]

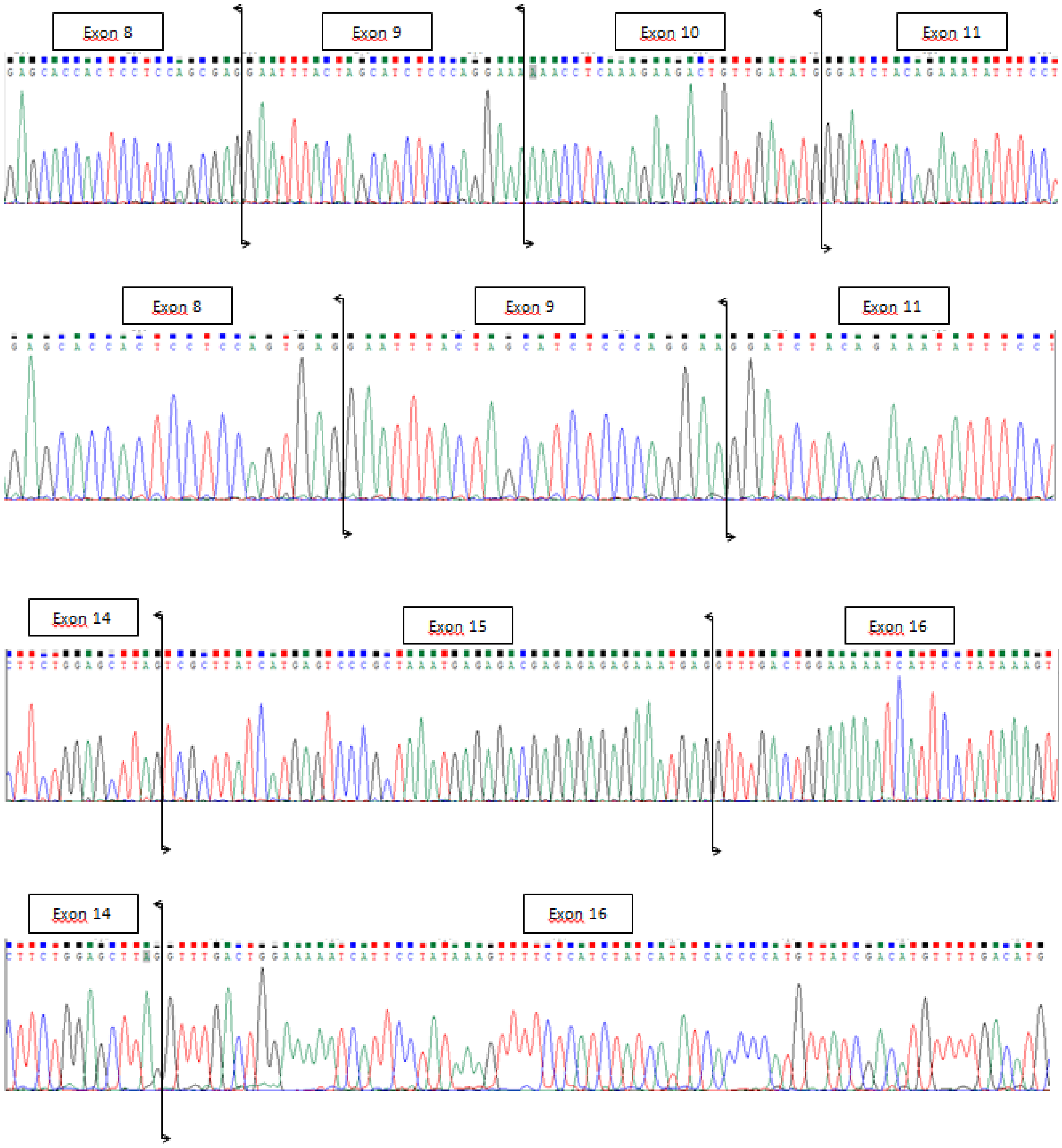

A

B

C

```
gtattttctgaataggaatattcattttgctgtcattcatccattctatattttgcagTC
----------------------------------------a-------------------

GCTTATCATGAGTCCCGCTAAATGAGAGACGAGAGAGAGAAATGAGgtactgacagaact   Equus asinus, caballus, and quagga
------------------------------------------------------------   Equus przewalskii
```

Figure S6. Results of *CSN1S2* II cDNA sequencing. (**A**) Alternative skipping of exon 10. (**B**) Altenative skipping of exon 15; the large arrows indicate exons. (**C**): Comparison of *Equus asinus* (GeneBank PSZQ01005937.1 from 24169059 to 24169178, complement), *Equus quagga* (GeneBank JAKJSB010001568.1 from 100955081 to 100954962, complement), *Equus caballus* (GeneBank PJAA01000004.1 from 66663354 to 66663473, complement), and *Equus przewalskii* (GeneBank ATBW01081756.1 from 33803 to 33922, complement) genomic sequences covering exon 15 and flanking regions of the *CSN1S2* II gene. Exon sequences are in uppercase and bold letters. Acceptor and donor splice sites are underlined and shaded. Dashes represent identical nucleotides to those in the upper lines. Alignment was performed using DNAsis pro Software v2.0 (Hitachi);
