# Supplementary material for: A Comprehensive Analysis of CSN1S2 I and II Transcripts Reveals Significant Genetic Diversity and Allele-Specific Exon Skipping in Ragusana and Amiatina Donkeys
Source: Animals (Basel). 2024 Oct 10;14(20):2918. doi: 10.3390/ani14202918 (PMC11503821; doi:10.3390/ani14202918)
Supplement: Supplementary file 1 [file animals-14-02918-s001.zip › Figure S7.pdf]

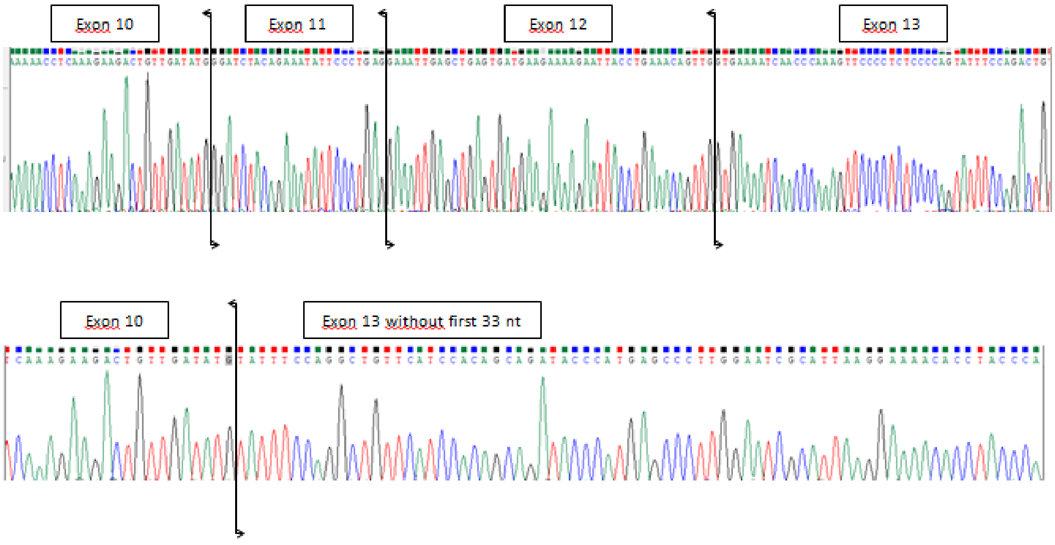

A

```
                           V  K  I  N  P  K  F  P  S  P  Q
ttattaataattttatttctttctagGTGAAAATCAACCCAAAGTTCCCCTCTCCCCAGT
----------------------------------------G-------------------

Y  F  Q  A  V  H  P  Q  Q  I  P  M  S  P  W  N  R  I  K  E
ATTTCCAGGCTGTTCATCCACAGCAGATACCCATGAGCCCTTGGAATCGCATTAAGGAAA
------------------------------------------------------------

N  T  Y  P  F  I  I  T  L
ACACCTACCCATTTATTATCACTTTGgtatgtttgctttttttaagtaacaaagttata  Equus asininus, caballus, przewalskii
------------------------------------------------------------  Equus quagga
```

B

Figure S7. (**A**) Results of *CSN1S2* II cDNA sequencing. Alternative skipping of exons 11 and 12 and of the first 33 nucleotides of exon 13. The large arrows indicate exons. (**B**) Comparison of *Equus asinus* (GeneBank PSZQ01005937.1 from 24166892 to 24167071), *Equus caballus* (GeneBank PJAA01000004.1 from 66661191 to 66661370), *Equus quagga* (GeneBank JAKJSB010001568.1 from 100957247 to 100957068, complement), and *Equus przewalskii* (GeneBank ATBW01081756.1 from 31501 to 31680) genomic sequences covering exon 13 and flanking regions of the *CSN1S2* II gene. Exon sequences and amino acids are in uppercase and bold letters. Canonical acceptor and donor splice sites and cryptic acceptor sites are underlined and shaded. Dashes represent identical nucleotides to those in the upper lines. Alignment was performed using DNAsis pro Software v2.0 (Hitachi).
