# Supplementary material for: A Comprehensive Analysis of CSN1S2 I and II Transcripts Reveals Significant Genetic Diversity and Allele-Specific Exon Skipping in Ragusana and Amiatina Donkeys
Source: Animals (Basel). 2024 Oct 10;14(20):2918. doi: 10.3390/ani14202918 (PMC11503821; doi:10.3390/ani14202918)
Supplement: Supplementary file 1 [file animals-14-02918-s001.zip › Figure S8.pdf]

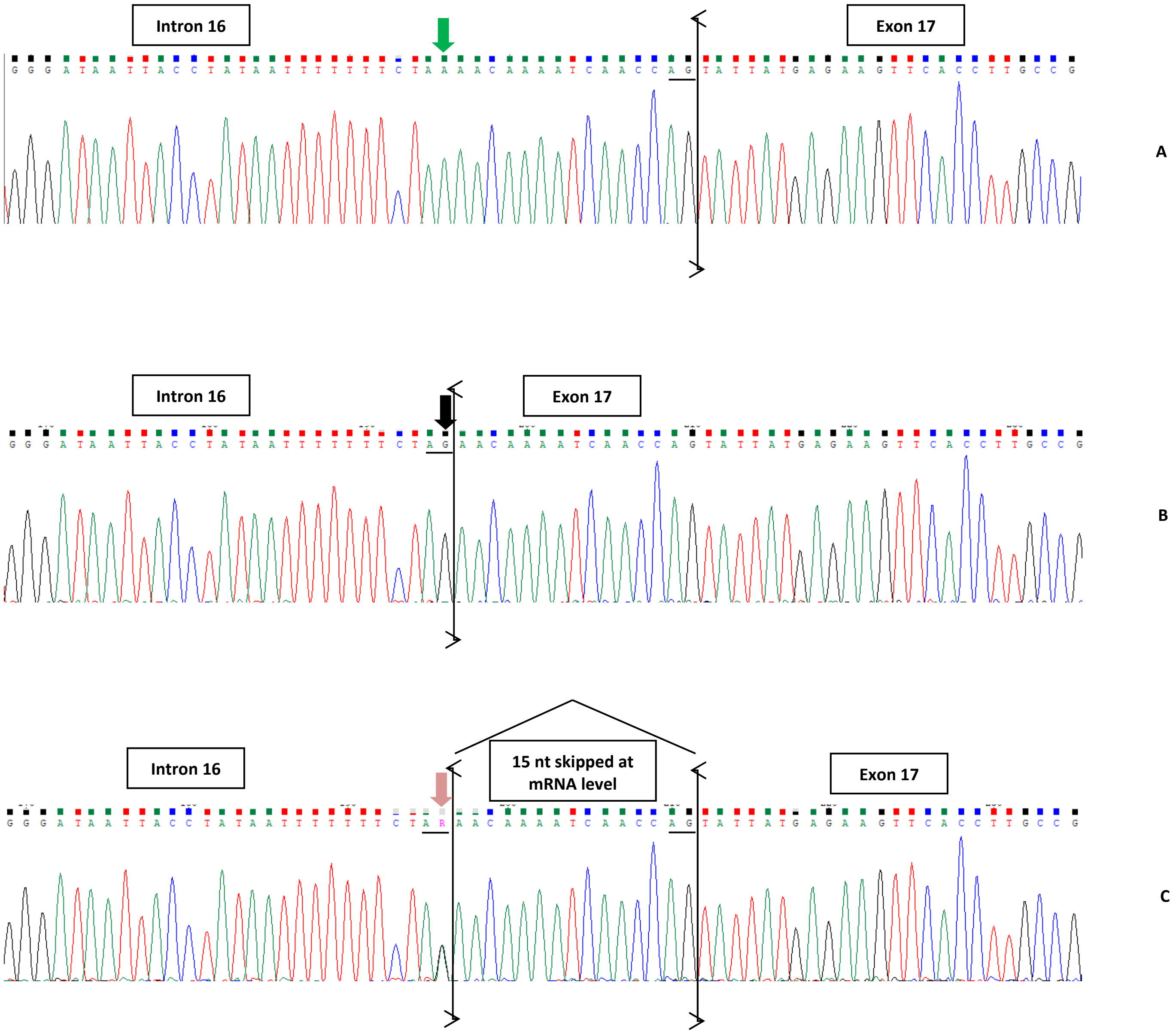

Figure S8. Results of the DNA sequencing of exon 17 and flanking regions of the donkey *CSN1S2 I* gene. (**A**) A/A homozygous sample. (**B**) G/G homozygous sample. (**C**) heterozygous sample. The acceptor splice sites of exon 17 are underlined. Solid arrows indicate the SNP (transition FM946022.1:c.375-1G>A) located in the acceptor splice site.
