# Supplementary material for: A Comprehensive Analysis of CSN1S2 I and II Transcripts Reveals Significant Genetic Diversity and Allele-Specific Exon Skipping in Ragusana and Amiatina Donkeys
Source: Animals (Basel). 2024 Oct 10;14(20):2918. doi: 10.3390/ani14202918 (PMC11503821; doi:10.3390/ani14202918)
Supplement: Supplementary file 1 [file animals-14-02918-s001.zip › Figure S9.pdf]

```
   Y Y E K F T L P Q Y F K I V H Q H Q T T M D P Q S H S K T N S Y Q I I P V L
aaaacaaaatcaaccagTATTATGAGAAGTTCACCTTGCCGCAATATTTCAAGATTGTTCATCAACACCAGACAACTATGGATCCACAGAGTCACAGTAAGACAAATTCTTACCAAATTATCCCCGTTCTGgt 1

   N K I N Q Y Y E K F T L P Q Y F K I V H Q H Q T T M D P Q S H S K T N S Y Q I I P V L
agAACAAAATCAACCAGTATTATGAGAAGTTCACCTTGCCGCAATATTTCAAGATTGTTCATCAACACCAGACAACTATGGATCCACAGAGTCACAGTAAGACAAATTCTTACCAAATTATCCCCGTTCTGgt 2

   Y Y E K F T L P Q Y F K I V R Q H Q T T M D P R S H S K T N S Y Q I I P V L
aaaacaaaatcaaccagTATTATGAGAAGTTCACCTTGCCGCAATATTTCAAGATTGTTCGTCAACACCAGACAACTATGGATCCACGGAGTCACAGTAAGACAAATTCTTACCAAATTATCCCTGTTCTGgt 3

   Y Y E K F T L P Q Y F K I V R Q H Q T T M D P R S H R K T N S Y Q I I P V L
aaaacaaaatcaaccagTATTATGAGAAGTTCACCTTGCCGCAATATTTCAAGATTGTTCGTCAACACCAGACAACTATGGATCCACGGAGTCACAGAAAGACAAATTCTTACCAAATTATCCCTGTTCTGgt 4

   Y Y Q K F T L P Q Y L K I V R Q   Q T T M N P W S H I K T N A Y Q V I P F L
aaaacaaaatcaaacagTATTATCAGAAGTTCACCTTGCCGGAATATCTCAAGATTGTTCGTCAA   CAGACAACTATGAATCCGTGGAGTCACATAAAGACAAATGCTTACCAAGTTATCCCTTTTCTGgt 5

   Y Y Q K F T L P Q Y L K I V R Q   Q T T L N P W S H I K T N A Y Q V I P F L
aaaacaaaatcaaacagTATTATCAGAAGTTCACCTTGCCGGAATATCTCAAGATTGTTCGTCAA   CAGACAACTTTGAATCCGTGGAGTCACATAAAGACAAATGCTTACCAAGTTATTCCTTTTCTGgt 6

   Y Y Q K F T L P Q Y L K I V R Q   Q T T M N P W S T I K T N A Y Q V I P F L
aaaacaaaatcaaacagTATTATCAGAAGTTCACCTTGCCGGAATATCTCAAGATTGTTCGTCAA   CAGACAACTATGAATCCGTGGAGTTACATAAAGACAAATGCTTACCAAGTTATCCCTTTTCTGgt 7

   Y Y Q K F T L P Q Y L K I V R Q   Q T T M N P W N H I K T N A Y Q V I P F L
aaaacaaaatcaaacagTATTATCAGAAGTTCACCTTGCCGGAATATCTCAAGATTGTTCGTCAA   CAGACAACTATGAATCCATGGAATCACATAAAGACAAATGCTTACCAAGTTATTCCTTTTCTGgt 8

   N K I K Q Y Y Q K F T W P Q Y I K T V H Q K Q K A M K P W N H I K T N S Y Q I I P N L
agAACAAAATCAAACAATATTATCAGAAATTCACCTGGCCCCAGTACATCAAGACTGTTCATCAAAAGCAGAAAGCCATGAAGCCATGGAATCACATTAAGACAAACAGTTACCAAATTATCCCCAATTTGgt 9

   N K I Y Q Y Y Q T F L W P E Y L K T V Y Q Y Q K T M T P W N H I K V K A Y Q I I P N L
agAACAAAATCTACCAATATTATCAGACATTCCTCTGGCCAGAGTATCTCAAGACTGTTTATCAATATCAGAAAACTATGACTCCATGGAATCACATCAAGGTAAAAGCTTACCAAATTATTCCCAATTTGgt 10

   K K I S Q R Y Q K F A L P Q Y L K T V Y Q H Q K A M K P W I Q P K T K       V I P Y V
agAAAAAAATCAGCCAGCGTTACCAGAAATTCGCCTTGCCCCAGTATCTCAAGACTGTTTATCAGCATCAGAAAGCTATGAAGCCATGGATTCAACCTAAGACAAAGG          TTATTCCCTATGTGgt 11

   K K I S Q H Y Q K F A W P Q Y L K T V Y Q Y Q K A M K P W T Q P K T N       V I P Y V
agAAAAAAATCAGCCAGCATTACCAGAAATTCGCCTGGCCCCAGTATCTCAAGACTGTTTATCAGTATCAGAAAGCTATGAAGCCATGGACTCAACCTAAGACAAACG          TTATTCCCTATGTGgt 12

   K K I S Q Y Y Q K F A W P Q Y L K T V D Q H Q K A M K P W T Q P K T N       A I P Y V
agAAAAAAATCAGCCAGTATTACCAGAAATTTGCCTGGCCCCAGTATCTCAAGACTGTTGATCAGCATCAGAAAGCTATGAAGCCATGGACTCAACCTAAGACAAATG          CTATTCCCTATGTGgt 13

   K K I S Q Y Y Q K F A W P Q Y L K T V D Q H Q K A M K P W T Q P K T N       A I P Y V
agAAAAAAATCAGCCAGTATTACCAGAAATTTGCCTGGCCCCAGTATCTCAAGACTGTTGATCAGCATCAGAAAGCTATGAAGCCATGGACTCAACCTAAGACAAACG          CTATTCCCTATGTGgt 14

   N K I N Q Y Y Q K L T W P Q Y L K T I S Q Y Q K T M K P W N H V K T N       V I P Y L
agAACAAAATCAACCAATATTATCAGAAATTAACCTGGCCCCAGTATCTCAAGACTATTTCTCAGTATCAGAAAACTATGAAGCCGTGGAATCACGTTAAGACAAATG          TTATCCCCTATCTGgt 15

   N K I S Q S Y Q K F T L P Q Y L K T V P P Y Q T T T K P W N Q I K I N A Y Q I I P I L
agAACAAAATCAGCCAATCTTATCAGAAGTTCACCCTGCCCCAATATCTTAAGACTGTTCCTCCATATCAGACAACAACGAAGCCATGGAATCAGATTAAGATAAATGCTTATCAAATTATCCCCATTCTGgt 16

   N K I N Q Y Y Q K F T L P Q Y L K T I H H Y Q T T M K P W N H V E I N A Y Q I I P V L
agAACAAAATCAACCAATATTATCAGAAGTTCACCTTGCCCCAATATCTCAAGACTATTCATCATTATCAGACAACAATGAAGCCATGGAATCACGTTGAGATAAATGCTTATCAAATTATCCCTGTTCTGgt 17

   N K I N Q Y Y Q K F T L P Q Y L K T V Q Q Y Q A A L K P W N H N T I N A Y Q I T P T L
agAACAAAATCAACCAATATTATCAGAAGTTCACCCTGCCCCAATATCTCAAGACTGTTCAGCAATATCAGGCAGCATTGAAACCATGGAATCACAATACGATAAATGCTTACCAAATTACCCCTACTCTGgt 18
```

Hippomorpha (1–4)
Ceratomorpha (5–8)
Perissodactyla (1–8)
Suiforme (9)
Tylopoda (10)
Ruminantia (11–14)
Cetancodonta (15)
Cetartiodactyla (9–15)
Euungulata (1–15)
Caniformia (16–17)
Feliformia (18)
Carnivora (16–18)
Ferae (16–18)
Ferungulata (1–18)

Figure S9. Comparison of genomic sequences covering exon 17 of the donkey *CSN1S2* I gene and predicted amino acid sequences with corresponding sequences from representative species of the *Perissodactyla*, *Cetartiodactyla* and *Carnivora* orders. Exon sequences are in uppercase and bold letters. Amino acid sequences are highlighted. Canonical and cryptic acceptor and donor splice sites are shaded. Alignment was performed using DNAsis pro Software v2.0 (Hitachi).

1: *Equus quagga* (GeneBank JAKJSB010001568.1 from 100893745 to 100893503); *Equus* asinus (present work); **2**: *Equus asinus* (present work, JREZ01000259.1 from 30185 to 30317, complement, and JADWZW020000003.1 from 152933647 to 152933779; **3**: *Equus caballus* (ATDM01077230.1 from 2431 to 2670, complement); **4:** *Equus przewalskii* (NW_007680800.1 from 1318 to 1080, complement); **5**: *Diceros bicornis* (JAQIGC010000012.1 from 27011816 to 27011576, complement); **6**: *Dicerorhinus sumatrensis* (JABWHU010001778.1 from 19044064 to 19043830, complement); **7**: *Ceratotherium simum* (PVLE01035877.1 from 14140 to 14314); **8**: *Tapirus indicus* (JAVSPQ010000004.1 from 54500678 to 54500912); **9**: *Sus scrofa* (SSNJ01000008.1 from 67019684 to 67019816, complement); **10**: *Camelus bactrianus* (OQ730238.1 from 12102 to 12234; *Camelus dromedarius* (OQ730239.1 from 12141 to 12273; *Camelus ferus* (NC_045697.1 from 53323924 to 53324056, complement); *Vicugna pacos* (NW_021964157.1 from 54357009 to 54357141, complement); **11**: *Bos taurus* (M94327.1 from 15396 to 15519); *Bos mutus* (NW_005394461.1 from 57201 to 57324); *Bos indicus* (NC_032655.1 from 88507513 to 88507636, complement); *Bison bison bison* (NW_011494880.1 from 1245318 to 1245441); **12**: *Bubalus bubalis breed Mediterranean* (MW159135.1 from 14035 to 14158); *Bubalus depressicornis* (JAMXBS010059713.1 from16389 to 16512); *Bubalus carabanensis* (JARFXY010000007.1 from 89755787 to 89755910); **13**: *Capra hircus* (JXYW01067838.1 from 11420 to 11543, complement); *Capra aegagrus* (JXYW01067838.1 from 11420 to 11543, complement); **14:** *Ovis aries* (JAWMPZ010000006.1 from 91401745 to 91401868, complement); **15**: *Balaenoptera* (VNFC03000009.1 from 53004866 to 53004743, complement); **16**: *Ailuropoda melanoleuca* (LNAT02000011.1 from 56029028 to 56028896, complement); **17**: *Canis lupus familiaris* (VSDE02000029.1 from 72117 to 72249 complement); **18**: *Felis catus* (NC_058371.1 from 148585412 to 148585544, complement); *Panthera pardus* (NW_026526688.1 from 148477160 to 148477292, complement); *Puma concolor* (NW_020339223.1 from 57708872 to 57709004, complement).
