# Supplementary material for: A Comprehensive Analysis of CSN1S2 I and II Transcripts Reveals Significant Genetic Diversity and Allele-Specific Exon Skipping in Ragusana and Amiatina Donkeys
Source: Animals (Basel). 2024 Oct 10;14(20):2918. doi: 10.3390/ani14202918 (PMC11503821; doi:10.3390/ani14202918)
Supplement: Supplementary file 1 [file animals-14-02918-s001.zip › Table S1.pdf]

Table S1. Oligonucleotide primers used for the characterization of the donkey *CSN1S2* I and *CSN1S2* II cDNAs.

Gene	Position (nt)	Primers sequence (5'–3')	GeneBank Acc. No.	Amplified DNA region
CSN1S1 I	7 - 27<br>Complementary to: 903 - 885	* Forward: CACTGCCTGCACTTTCTTGTC<br>* Reverse: GGCTTGATGTTTCGGCCTT	FM946022.1	From exon 1 (partial) to exon 19 (partial)
CSN1S1 II	13 - 35<br>Complementary to: 852 - 831	* Forward: AGTTCTCCAGTATTCCACTTGGG<br>* Reverse: CGGAAACAGCACTGAAAAGACT	FN298386.2	From exon 1 (partial) to exon 16 (partial)
CSN1S1 I	152933458 - 152933477<br>Complementary to: 152934015 - 152933994	** Forward: TCTCAGTTCTACAGTTCATT<br>** Reverse: TTAAGTCCAATGTAAATCCAAT	JADWZW020000003.1	Exon 17 and flanking regions

* Amplification and cloning of the *CSN1S2* I and *CSN1S2* II cDNAs.
** DNA sequencing and *Xba*I PCR-RFLP genotyping assay for the *CSN1S2* I.
