# Supplementary material for: A Comprehensive Analysis of CSN1S2 I and II Transcripts Reveals Significant Genetic Diversity and Allele-Specific Exon Skipping in Ragusana and Amiatina Donkeys
Source: Animals (Basel). 2024 Oct 10;14(20):2918. doi: 10.3390/ani14202918 (PMC11503821; doi:10.3390/ani14202918)

Table S2. Detection of polymorphisms at *CSN1S2* I locus in Ragusana donkeys and corresponding electropherograms.

Exons	Location	SNP	aa change in the full-length peptide chain	Electropherogram
**8**	19	ACA>GCA	p.T73>A	A
**10**	15	TTT>TTC	p.F88	B
**12**	7	ATC>GTC	p.I109>V	C
	70	ATT>GTT	p.I130>V	D
	119	ATT>ACT	p.I146>T	E
**14**	12	AAG>AAA	p.K162	F
**17**	79	GAT>TAT	p.D217>Y	G

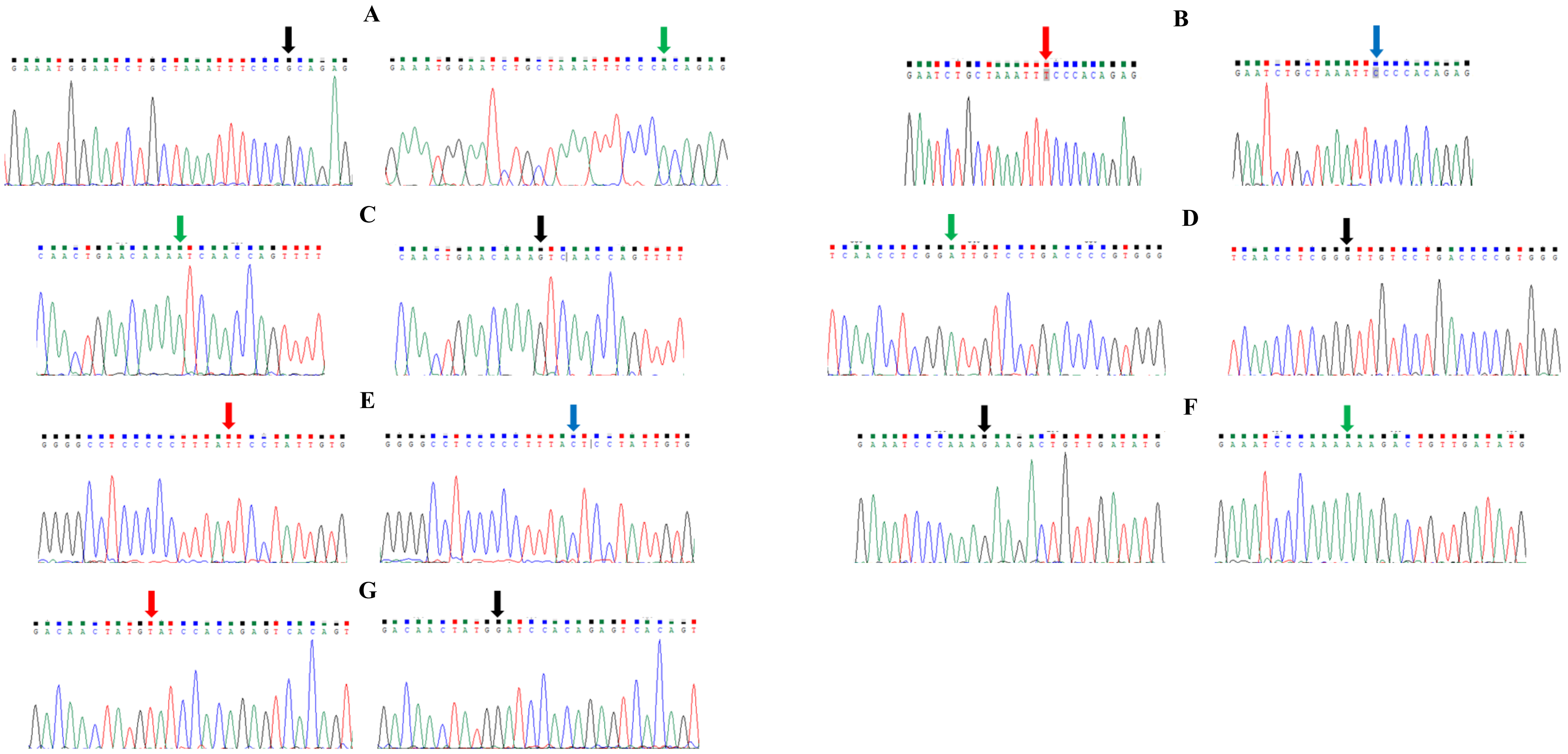

Supplement: Supplementary file 1 [file animals-14-02918-s001.zip › Table S2.pdf]
