# Supplementary material for: A Comprehensive Analysis of CSN1S2 I and II Transcripts Reveals Significant Genetic Diversity and Allele-Specific Exon Skipping in Ragusana and Amiatina Donkeys
Source: Animals (Basel). 2024 Oct 10;14(20):2918. doi: 10.3390/ani14202918 (PMC11503821; doi:10.3390/ani14202918)

Table S3. Polymorphisms detected at *CSN1S2* II cDNA in Ragusana donkeys, corresponding electropherograms, and comparison with counterpart sequences available in the literature and GeneBank.

Exons	Location SNP	Mutation present work	aa change in the full-length peptide chain	Auzino et al. (2022)	FN298386	XM_044766711.1	PSZQ01005937.1	Electropherogram
**7**	16	CTC>TTC	p.L63>F	CTC	CTC	CTC	CTC	A
**8**	15	CAC>CAA	p.H70>Q	CAC	CAC	CAC	CAC	B
	24	AGC>AGT	p.73S	AGC	AGC	AGC	AGC	C
**10**	22	GAT>AAT	p.D90>N	GAT	GAT	GAT	GAT	D
**11**	18	TTT>TTC	p.97F	TTC	TTT	TTC	TTC	E
**13**	43	GCT>ACT	p.A129>T	GCT/ACT	GCT	GCT	TCT (p.129S)	F
	58	CAT>TAT	p.H131>Y	CAT	CAT	CAT	CAT	G
	86	ATT	p.I142	ATT	AGT (p.S142)	ATT	ATT	H
	92	GAA>GGA	p.E144>G	GAA	GAA	GAA	GAA	I
**14**	8	TTT>TCT	p.F157S	TTT	TTT	TTT	TTT	L

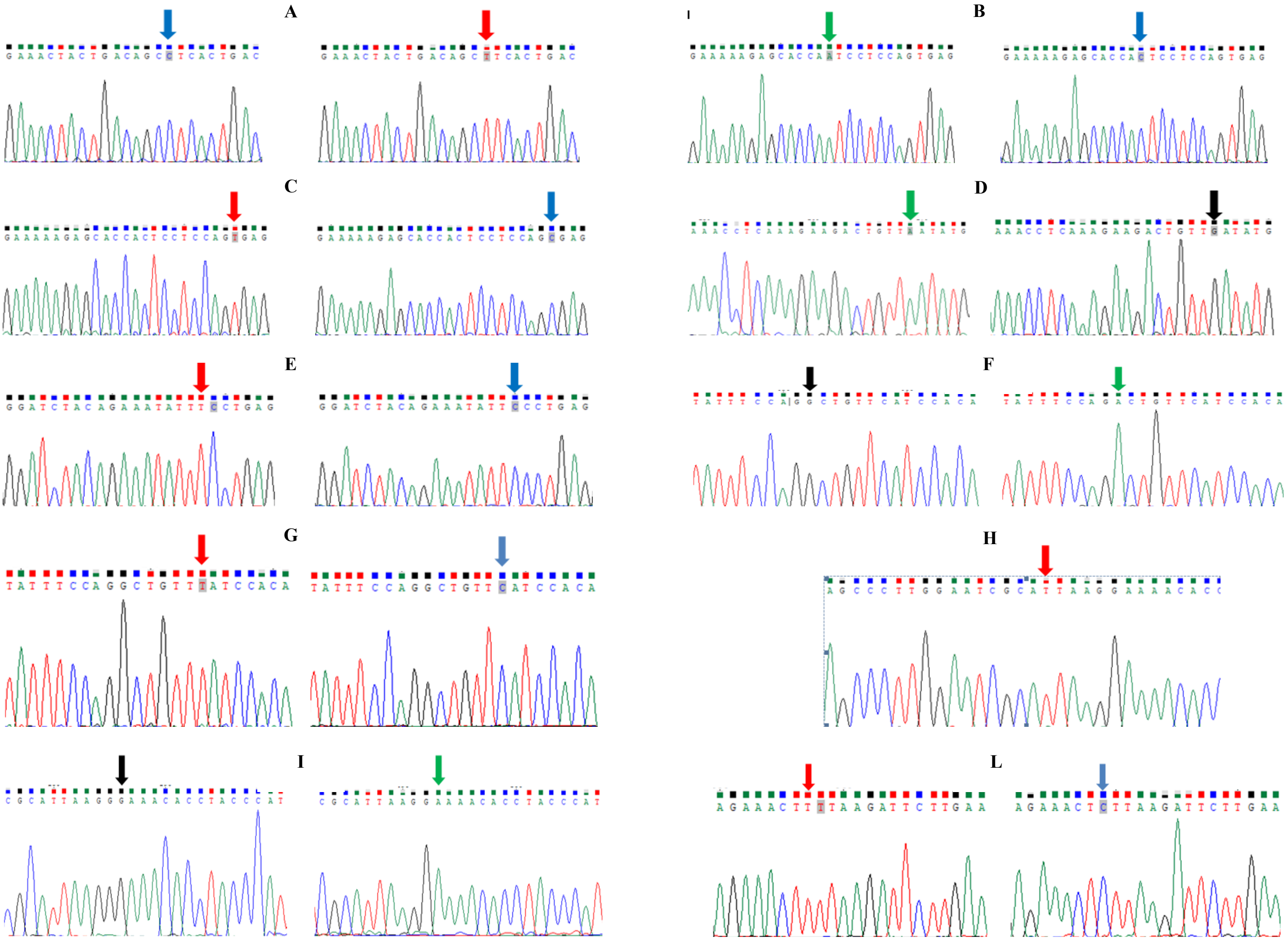

Supplement: Supplementary file 1 [file animals-14-02918-s001.zip › Table S3.pdf]
