# Supplementary material for: A Comprehensive Analysis of CSN1S2 I and II Transcripts Reveals Significant Genetic Diversity and Allele-Specific Exon Skipping in Ragusana and Amiatina Donkeys
Source: Animals (Basel). 2024 Oct 10;14(20):2918. doi: 10.3390/ani14202918 (PMC11503821; doi:10.3390/ani14202918)
Supplement: Supplementary file 1 [file animals-14-02918-s001.zip › Table S4.pdf]

Table 4S. Genotyping data and allele frequency of the FM946022.1:c.375-1G>A transition at the acceptor splice site of exon 17 in the *CSN1S2* I gene in Ragusana and Amiatina donkeys.

	**Genotype Distribution**			**Total**	**Total Allelic Frequency**	
	**AA**	**AG**	**GG**		**A**	**G**
Obs Ragusana	6	41	58	105	0.2437	0.7563
Obs Amiatina	-	5	9	14		
Obs Tot	6	46	67	119		
Exp Tot	7.0672	43.8655	68.0672			
$\chi^2 = 0.2818$						
$P \leq 0.05$						
d.f.=1						
